# Parkinson’s VPS35[D620N] mutation induces LRRK2-mediated lysosomal association of RILPL1 and TMEM55B

**DOI:** 10.1126/sciadv.adj1205

**Published:** 2023-12-13

**Authors:** Prosenjit Pal, Matthew Taylor, Pui Yiu Lam, Francesca Tonelli, Chloe A. Hecht, Pawel Lis, Raja S. Nirujogi, Toan K. Phung, Wondwossen M. Yeshaw, Ebsy Jaimon, Rotimi Fasimoye, Emily A. Dickie, Melanie Wightman, Thomas Macartney, Suzanne R. Pfeffer, Dario R. Alessi

**Affiliations:** ^1^ MRC Protein Phosphorylation and Ubiquitylation Unit, School of Life Sciences, University of Dundee, Dundee DD1 5EH, UK.; ^2^ Aligning Science Across Parkinson’s (ASAP) Collaborative Research Network, Chevy Chase, MD 20815, USA.; ^3^ Department of Biochemistry, Stanford University School of Medicine, Stanford, CA 94305-5307, USA.

## Abstract

We demonstrate that the Parkinson’s VPS35[D620N] mutation alters the expression of ~220 lysosomal proteins and stimulates recruitment and phosphorylation of Rab proteins at the lysosome. This recruits the phospho-Rab effector protein RILPL1 to the lysosome where it binds to the lysosomal integral membrane protein TMEM55B. We identify highly conserved regions of RILPL1 and TMEM55B that interact and design mutations that block binding. In mouse fibroblasts, brain, and lung, we demonstrate that the VPS35[D620N] mutation reduces RILPL1 levels, in a manner reversed by LRRK2 inhibition and proteasome inhibitors. Knockout of RILPL1 enhances phosphorylation of Rab substrates, and knockout of TMEM55B increases RILPL1 levels. The lysosomotropic agent LLOMe also induced LRRK2 kinase–mediated association of RILPL1 to the lysosome, but to a lower extent than the D620N mutation. Our study uncovers a pathway through which dysfunctional lysosomes resulting from the VPS35[D620N] mutation recruit and activate LRRK2 on the lysosomal surface, driving assembly of the RILPL1-TMEM55B complex.

## Teaser

A pathway linking VPS35 and LRRK2 with the lysosomal dysfunction is described revealing TMEM55B as a LRRK2 network component.

## INTRODUCTION

Mutations that increase the kinase activity of the leucine-rich repeat kinase-2 (LRRK2) represent one of the most common inherited causes of Parkinson’s disease (PD) (*1**,* *2*) and have also been linked to inflammatory bowel disease (*3**,* *4*). LRRK2 is a large 2527-residue multidomain protein consisting of two catalytic domains: A Roco type guanosine triphosphatase (GTPase) in addition to a protein kinase (*5*). LRRK2 phosphorylates a subgroup of Rab GTPase proteins (Rab1, Rab3, Rab8, Rab10, Rab12, Rab29, Rab35, and Rab43) (*6**,* *7*) that coordinate membrane homeostasis and endocytic and exocytic pathways (*8*). LRRK2 phosphorylates Rab substrates at a conserved Ser or Thr site lying at the center of the effector-binding switch-II motif (Thr^72^ for Rab8A, Thr^73^ for Rab10, and Ser^106^ for human Rab12) (*6**,* *7*). This reaction is counteracted by the PPM1H phosphatase that efficiently dephosphorylates Rab proteins (*9**,* *10*). LRRK2 phosphorylation of Rab proteins affects their ability to interact with its cognate effectors, for example, phosphorylation of Rab8 blocks interactions with Rabin-8 [a guanosine diphosphate (GDP)/guanosine triphosphate (GTP) exchange factor] and GDI (GDP dissociation inhibitor) that shuttles Rab proteins between membranes (*6**,* *7*).

Four scaffolding proteins, namely, RILPL1, RILPL2, JIP3, and JIP4, interact specifically with LRRK2 phosphorylated Rab8A and Rab10 with higher affinity than dephosphorylated Rab proteins (*6**,* *11**,* *12*). This interaction is mediated by an α-helical RH2 motif having conserved basic residues that form ionic interactions with the LRRK2 phosphorylated switch-II motif residue (*11*). The interaction of RILPL1 with phosphorylated Rab proteins interferes with ciliogenesis (*6**,* *13**,* *14*) and, in cholinergic neurons in the striatum, leads to disruption of a Sonic hedgehog neuroprotective circuit that supports dopaminergic neurons, providing a pathway by which LRRK2 may be linked to PD pathology (*13*).

Rab proteins also play a key role in controlling LRRK2 kinase activity by binding to the N-terminal ARM domain and recruiting LRRK2 to membranes where it becomes activated (*15**,* *16*). Recent work has pinpointed three Rab binding sites within the LRRK2 ARM domain. Site 1 binds to dephosphorylated Rab29, Rab32, as well as Rab8 and possibly Rab10 (*17**–**19*). Site 2 interacts specifically to LRRK2-phosphorylated Rab8 and Rab10 in a feed-forward pathway that drives membrane recruitment and activation of LRRK2 (*18*). Site 3 interacts with Rab12 (*20*), and ablation of this site or knockout (KO) of Rab12 has the largest effect in regulating the basal activity of LRRK2 as judged by its ability to phosphorylate Rab10 (*20**,* *21*).

Lysosomal dysfunction is strongly associated with PD (*22**,* *23*). *LRRK2* and other PD-associated genes, including *GBA1* (*24*), *ATP13A2* (*25*), and *TMEM175* (*26*), play a critical role in controlling lysosome homeostasis and function. Elevated LRRK2 kinase activity reduces lysosomal degradative activity and autophagic flux in a manner that is counteracted by LRRK2 inhibitors (*27**–**30*). Furthermore, damage of lysosomes following infection (*31*) or treatment with agents such as l-leucyl-l-leucine methyl ester (LLOMe) induces recruitment of LRRK2 to lysosomal membranes (*12**,* *32*). At the lysosome, LRRK2 is activated and found to phosphorylate Rab proteins, thereby recruiting JIP4, which promotes formation of tubular structures that release membranous content from lysosomes (*12*). Recent work reveals that LRRK2 negatively regulates lysosomal degradative activity in macrophages and microglia via a transcriptional mechanism involving transcription factor E3 (TFE3) (*33*). Depletion of LRRK2 and inhibition of LRRK2 kinase activity both enhance lysosomal proteolytic activity and increase the expression of multiple lysosomal hydrolases (*33*). Other work has revealed that LRRK2 kinase activity controls PD relevant lipids such as bis(monoacylglycerol)phosphates as well as glycosphingolipids at the lysosome (*34**,* *35*).

The VPS35 component of the retromer complex transports select endosomal cargo proteins between endosomal compartments and the Golgi and has been linked to Parkinson’s (*36**,* *37*) as well as Alzheimer’s diseases (*38*). The D620N mutation in VPS35 causes autosomal dominant PD and stimulates the LRRK2 pathway via an unknown mechanism (*39*). VPS35[D620N] knock-in cells and tissues display markedly enhanced Rab phosphorylation to a higher level than observed with LRRK2 PD pathogenic mutations (*39**,* *40*), and this mutation has been proposed to lead to lysosomal dysfunction (*41**,* *42*). It is possible that D620N VPS35 disruption of selective endosomal cargo trafficking triggers lysosomal dysfunction, thereby activating LRRK2.

In this study, we sought to investigate the impact that elevated LRRK2 signaling has on lysosomal protein content using LysoTag immunoprecipitation (IP) coupled to mass spectrometry (MS). We have uncovered a pathway by which lysosomal stress or dysfunction resulting from the VPS35[D620N] mutation or the lysosomotropic agent LLOMe induces lysosomal recruitment of LRRK2, resulting in phosphorylation of Rab proteins, which triggers recruitment of RILPL1. Our data suggest that RILPL1 then interacts via its conserved C-terminal region with a conserved domain of TMEM55B, an integral lysosomal membrane protein. Our data provide insights into a pathway by which LRRK2 communicates with the lysosome.

## RESULTS

### VPS35[D620N] mutation alters the lysosomal protein content

**Fig. 1.**
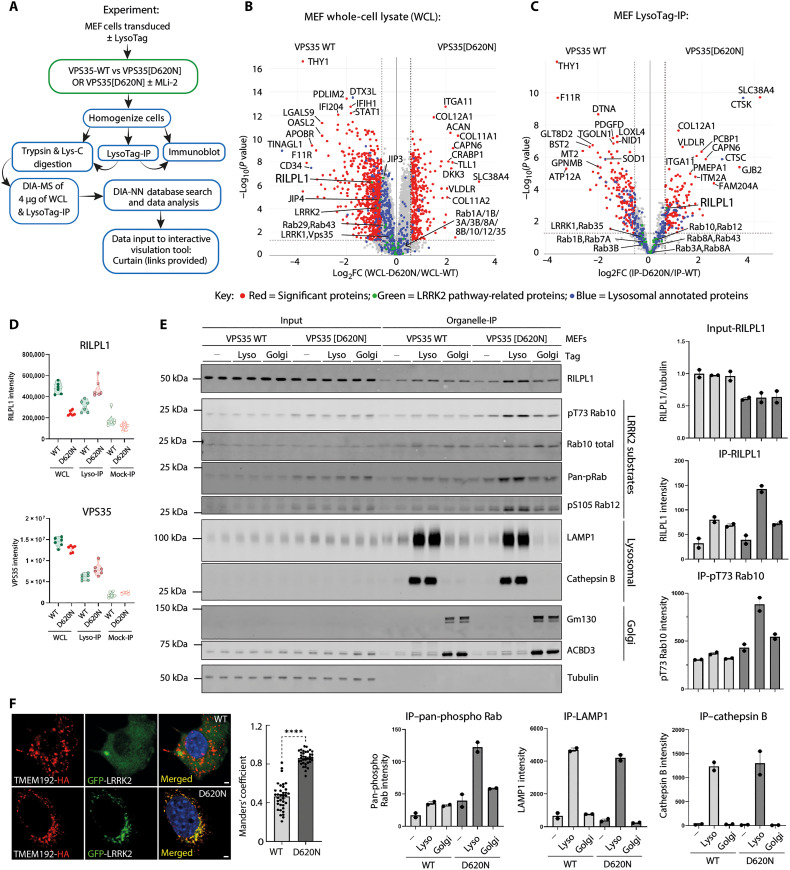
Phospho-Rab and RILPL1 enrichment at VPS35[D620N] mutant lysosome. (**A**) Workflow of the LysoTag IP methodology. (**B** and **C**) The indicated littermate-matched MEFs were transduced ± LysoTag (TMEM192-3xHA) and subjected to LysoTag IP. A sample of the homogenate was removed and designated WCL. Experiments were performed in six technical replicates and analyzed by DIA-MS, and the data are presented as Volcano plots (WCL Curtain link: https://curtain.proteo.info/#/0e673d58-d8f2-4368-996b-0869d5513d46 and lysosome Curtain link:https://curtain.proteo.info/#/0e673d58-d8f2-4368-996b-0869d5513d46 and table S4). The red dots represent the substantially differentiated proteins with fold change > 1.5 and *P* < 0.05, the green dots represent the LRRK2 pathway–related proteins, and the blue dots represent the lysosomal annotated proteins. (**D**) Violin plots of RILPL1 and VPS35 levels derived from experiment in (B) and (C). (**E**) Indicated MEFs transduced ± either the LysoTag (TMEM192-3xHA) or GolgiTag (TMEM115-3xHA) were subjected to organelle isolation as in (C) and (D). Two micrograms of both the immunoprecipitate and respective input (WCL) was subjected to immunoblot analysis using the LI-COR Odyssey CLx Western blot imaging and the indicated antibodies. Each lane indicates a sample derived from a different dish of cells. Quantitation of immunoblotting data (performed using ImageStudioLite software version 5.2.5, RRID:SCR_013715) is shown as mean ± SEM. (**F**) Indicated homozygous knock-in (KI) LysoTag (TMEM192-3xHA)–transduced MEFs were transfected with GFP-LRRK2. Colocalization of GFP-LRRK2 with TMEM192-3xHA was quantified and analyzed using Manders’ coefficient after automatic thresholding. Data from 36 technical replicates (12 cells from three biological replicates) are presented as mean ± SEM. Statistical analysis was performed using two-tailed unpaired Student’s *t* test (****P < 0.0001). Scale bars, 2 μm.

To study the impact that the VPS35[D620N] mutation has on the lysosome, we first performed a LysoTag IP in lysates from littermate-matched wild-type (WT) and homozygous knock-in *VPS35[D620N]* mouse embryonic fibroblasts (MEFs) transduced to stably express LysoTag (TMEM192-3xHA) (*43*), using a workflow described in Fig. 1A. Immunoblot analysis confirmed previous data that the VPS35[D620N] mutation enhanced LRRK2-mediated Rab10 phosphorylation ~4-fold (fig. S1A). Data-independent acquisition (DIA) MS with equal protein amounts (4 μg) of whole-cell lysates (WCLs) (Fig. 1B) and isolated lysosomes were undertaken, with six replicates (Fig. 1C). MS data were searched through DIA-NN (*44*) and visualized using an interactive visualization tool called Curtain [https://curtain.proteo.info, 10.5281/zenodo.8138473; RRID: SCR_024465 (*45*)], in which the data can be analyzed using the web links provided in the figure legend. The experiments revealed that in WCLs, the D620N mutation altered expression >2-fold of 363 proteins, with 70 increasing (0.82%) and 293 decreasing (3.44%) (Fig. 1B and table S4). Similarly, for the LysoTag IP, 81 proteins increased and 136 decreased >2-fold (Fig. 1C; fig. S1, B to D; and table S4). Violin plots for the top 12 proteins whose levels increase with the D620N mutation (including cathepsin C, cathepsin K, SLC38A4, and GJB2) and the top 12 that decrease (including THY1, HSPA1A/B, MT2, and SFRP2) are shown in fig. S2 (A and B). We undertook gene ontology (GO) metascape analysis (*46*) of the proteins that were increased or decreased in VPS35[D620N] compared to WT MEFs in whole lysates (fig. S3A and table S4) and LysoTag immunoprecipitates (fig. S3B and table S4). In WCLs, proteins that changed in VPS35[D620N] MEFs affected a wide range of biology including response to virus, cell proliferation, adhesion, nucleosome, and extracellular matrix. The top proteins changing in the VPS35[D620N] lysosome immunoprecipitates included those affecting extracellular matrix, glycosaminoglycan binding, vacuolar membrane, transmembrane transport, and anion channel activity.

### LRRK2 activity drives the recruitment of RILPL1 to lysosomes in VPS35[D620N] cells

We searched the data for known LRRK2 pathway components and found one protein, the phospho-Rab effector protein RILPL1 (*6**,* *11*), whose levels were enriched in VPS35[D620N] compared to WT lysosomes (Fig. 1D). To investigate this further, we performed LysoTag (*43*) and GolgiTag (*47*) IPs in parallel from littermate-matched WT and homozygous VPS35[D620N] knock-in MEFs (Fig. 1E). This revealed that the VPS35[D620N] mutation enhanced recruitment of RILPL1 specifically to the lysosome, but not the Golgi (Fig. 1E). We also observed increased phosphorylation of Rab10 (Thr^73^) and Rab12 (Ser^105^), as well as phosphorylated Rab proteins detected using a pan phospho-specific Rab antibody, at the VPS35[D620N] lysosome, but not at the Golgi (Fig. 1E). Colocalization of LRRK2 with TMEM192 (a lysosomal membrane integral protein) indicated substantially enhanced (*P* < 0.0001) recruitment of LRRK2 to the lysosome in the VPS35[D620N] MEFs compared to the WT MEFs (Fig. 1F).

**Fig. 2.**
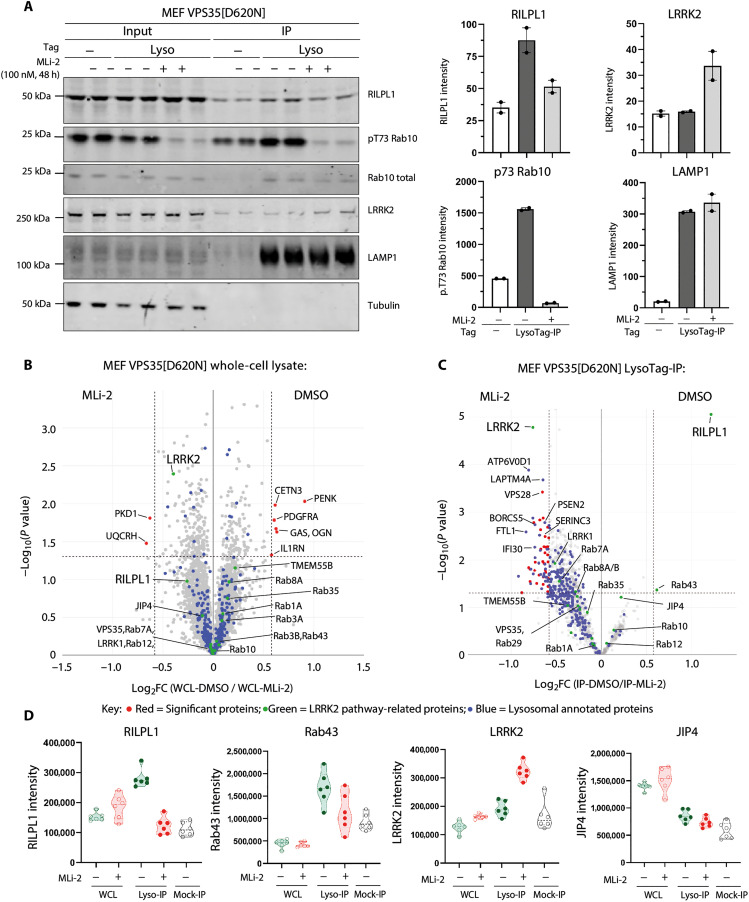
Lysosomal recruitment of RILPL1 is dependent on LRRK2 kinase activity. (**A**) *VPS35[D620N]* homozygous knock-in MEFs transduced ± LysoTag (TMEM192-3xHA) were subjected to ±100 nM MLi-2 treatment for 48 hours before homogenization. A sample of the homogenate was removed and labeled input, and the remainder were subjected to LysoTag IP. Two micrograms of both the input and immunoprecipitate was subjected to quantitative immunoblot analysis using the LI-COR Odyssey CLx Western blot imaging system and indicated antibodies. Each lane represents a sample derived from a different dish of cells. Quantitation of immunoblotting data (performed using ImageStudioLite software version 5.2.5, RRID:SCR_013715) is shown as mean ± SEM. (**B** and **C**) Immunoprecipitate and input (WCL) samples generated as in (A) were performed in six technical replicates and were subjected to DIA-MS analysis. The volcano plots show the proteome changes of MEFs D620N with dimethyl sulfoxide (DMSO) versus MLi-2 treatment in WCLs (Curtain link: https://curtain.proteo.info/#/d864df78-e2a5-4a64-99fb-8c5d8b2e1ab8) (B) and lysosomes (Curtain link: https://curtain.proteo.info/#/062e0a64-1fd3-4cc1-833a-a5b007d95a3c) (table S5) (C). Proteins with fold change of >1.5 compared to Mock-IP samples are highlighted in the volcano plot (fig. S2A). The red dots represent the notable differentiated proteins with fold change > 1.5 and *P* < 0.05, the green dots represent the LRRK2 pathway–related proteins, and the blue dots represent the lysosomal annotated proteins. (**D**) Violin plots of the levels of the indicated proteins.

We next undertook LysoTag IPs from VPS35[D620N] knock-in MEFs treated for 48 hours ± 100 nM MLi-2, a highly specific and well-characterized LRRK2 inhibitor (*48*). Immunoblotting revealed that MLi-2 reduced levels of RILPL1 at the VPS35[D620N] lysosome to background levels observed in the WT immunoprecipitate (Fig. 2A). As expected, MLi-2 also ablated Rab10 phosphorylation in whole-cell extract and the lysosome (Fig. 2A). MS analysis revealed that MLi-2 did not substantially alter the expression of any protein in the WCL >2-fold (Fig. 2B and table S5). For the LysoTag IP, RILPL1 was the clear-cut protein whose association with the lysosome was most reduced (~2.5-fold) by MLi-2 treatment (Fig. 2C, fig. S4, and table S5). Rab43, an LRRK2 substrate, was also reduced, although at borderline statistical significance (Fig. 2C). To our knowledge, Rab43 has not been associated with the lysosome; however, a recent report (*49*) suggests at least some RILPL1 localization to the lysosome. The lysosomal levels of several other proteins including LRRK2, ATP6V0D1, Laptm4a, and VPS28 were moderately increased (~1.5-fold) following MLi-2 treatment (Fig. 2C and fig. S4). We also investigated the levels of the top 24 proteins analyzed in fig. S2 whose expression was most affected by the VPS35[D620N] mutation in the LysoTag IP. We found that MLi-2 had little effect on most proteins; however, the MT2 membrane-anchored serine protease, whose lysosomal levels were reduced in the VPS35[D620N] lysosomes, were moderately increased following MLi-2 treatment (fig. S5, A and B, and table S5).

We next investigated the levels of the set of lysosomal proteins (cathepsin B, cathepsin C, cathepsin D, cathepsin L, GBA, LAMP1, TFE3 and TFEB) that were previously reported to increase following inhibition or depletion of LRRK2 in macrophages and microglia (*33*). In WT versus VPS35[D620N] and VPS35[D620N] ± MLi-2 MEF datasets, only cathepsin L was moderately decreased by the VPS35[D620N] mutation (fig. S5, C and D, and table S5). We observed an opposite response for levels of cathepsin C whose lysosomal levels were markedly increased by VPS35[D620N] compared to WT. The levels of cathepsin B, cathepsin D, GBA, LAMP1, and TFEB were not altered in VPS35[D620N] LysoTag IP MEFs. Although the levels of TFE3 transcription factor was moderately increased in the LysoTag IP of VPS35[D620N] MEFs, it should be noted that TFE3 and TFEB are not strongly enriched in the LysoTag IP compared to mock and WCLs (fig. S5C and table S5). These data suggest that transcriptional responses to LRRK2 activity are likely to be cell type specific. We found that there were no substantial changes in the core retromer complex components VPS26A, VPS26B, and VPS29 in WCL and LysoTag IP from the VPS35 WT, and D620N and D620N ± MLi-2 MEF datasets (fig. S6, A to D).

### LRRK2 activity reduces RILPL1 levels in whole-cell extracts

**Fig. 3.**
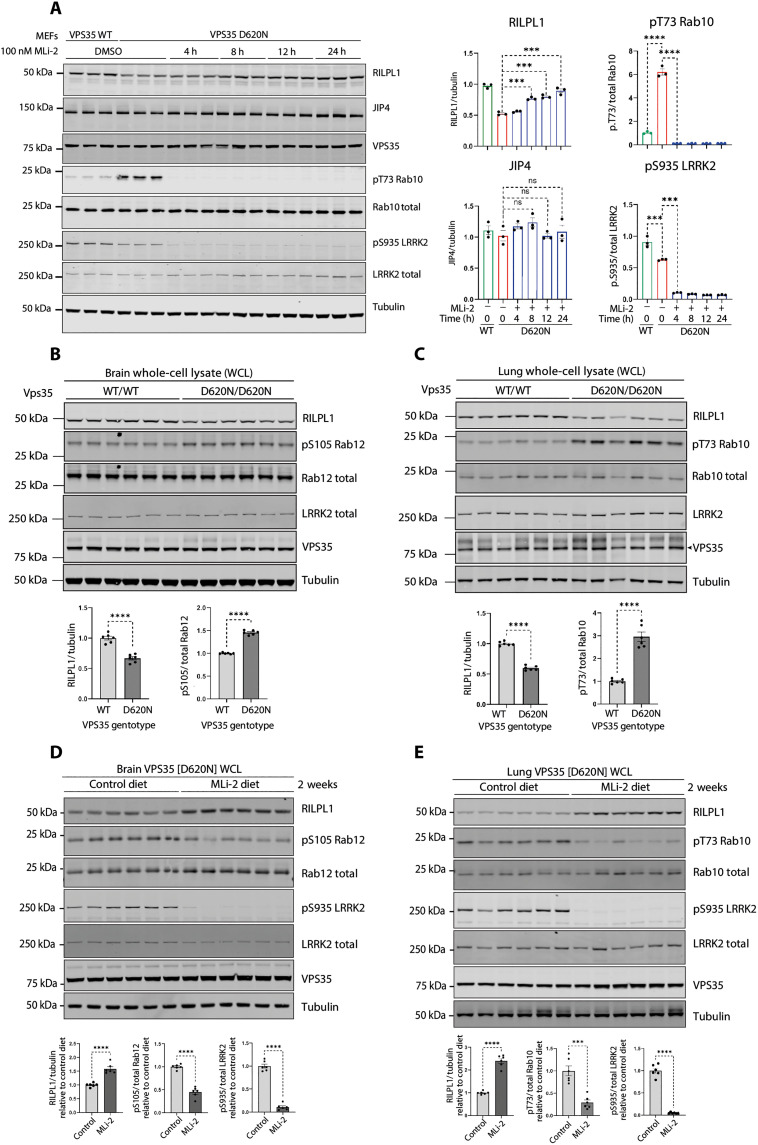
Enhanced LRRK2 activity by VPS35[D620N] mutation reduces expression of RILPL1. (**A**) Littermate-matched WT and *VPS35[D620N]* homozygous knock-in MEFs were treated ± 100 nM MLi-2 for the indicated times before lysis. Lysates were subjected to quantitative immunoblot analysis of the LI-COR Odyssey CLx Western blot imaging system and indicated antibodies. Technical replicates represent cell extract obtained from a different dish of cells. Quantitation of immunoblotting data (performed using ImageStudioLite software version 5.2.5, RRID:SCR_013715) is shown as mean ± SEM. Data were analyzed using two-tailed unpaired *t* test (**P < 0.01, ***P < 0.001, and ****P < 0.001). (**B**) Brain and (**C**) lung tissues were harvested from 4-month-old, littermate-matched WT and homozygous VPS35[D620N] knock-in mice. Twenty micrograms of whole tissue extract was subjected to immunoblot analysis using indicated antibodies as described in (A). (**D** and **E**) Four-month-old littermate-matched homozygous Vps35[D620N] knock-in mice were fed on either a control diet or MLi-2 diet for 2 weeks before tissue harvesting. Brain (D) and lung (E) tissue extracts were analyzed by immunoblotting, and quantification was performed as previously described in (A).

MS data of WCLs revealed that levels of RILPL1 were reduced in the VPS35[D620N] background, suggesting that recruitment of RILPL1 to the lysosome accelerates its degradation (Fig. 1, D and E). To explore whether this was linked to LRRK2 kinase activity, we treated VPS35[D620N] knock-in MEFs with MLi-2. Within 8 hours, RILPL1 levels returned to that of WT cells (Fig. 3A). Levels of RILPL1 in homozygous VPS35[D620N] mouse brain (Fig. 3B) and lung extracts (Fig. 3C) were also ~40% lower compared to WT. Analysis of tissues from VPS35[D620N] mice fed with a diet containing MLi-2 for a 2-week period revealed that inhibiting LRRK2 notably increased RILPL1 levels in WCLs, ~1.6-fold in the brain (Fig. 3D) and ~2.5-fold in the lung (Fig. 3E).

To establish whether VPS35[D620N]-mediated reduction in RILPL1 is mediated via lysosomal or proteasomal degradation, VPS35[D620N] MEFs were treated with either cycloheximide (50 μg/ml) (translation inhibitor) alone, or a combination of cycloheximide and 10 μM MG-132 (proteasome inhibitor), or a combination of cycloheximide and lysosomal protease inhibitor cocktails (5 μM E64D, 10 μM leupeptin, and 10 μM pepstatin A) for 8 and 12 hours before lysis. A moderate (*P* = 0.0116) recovery of RILPL1 levels was observed after 8 hours of cycloheximide + MG-132 treatment when compared to the cycloheximide treatment alone (fig. S7), whereas 12 hours of treatment showed substantial recovery (*P* = 0.0008). Combination of cycloheximide and lysosomal protease inhibitor cocktail treatment did not result in notable changes of RILPL1 levels, suggesting that the degradation of RILPL1 is mediated by the proteasome pathway.

### MLi-2 treatment reduces perinuclear lysosomes in VPS35[D620N] MEFs

**Fig. 4.**
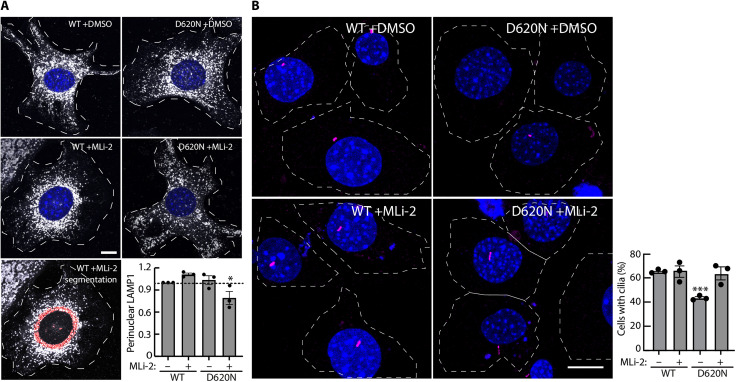
LRRK2 activity increases perinuclear lysosomes, and LRRK2 inhibition restores ciliogenesis in VPS35[D620N] MEFs. (**A**) LRRK2 activity increases perinuclear lysosomes in VPS35[D620N] MEFs. Indicated cell types were treated for 2 hours ± 200 nM MLi-2 or DMSO. Lysosomes were stained with anti-LAMP1 antibodies; nuclei were stained using 4′,6-diamidino-2-phenylindole (DAPI). Integrated intensity of perinuclear LAMP1 was quantified by measuring the intensity of LAMP1 staining within 20 pixels of the nucleus (see red labeled example, bottom left) and normalized for total LAMP1 staining. Error bars represent SEM from three independent experiments with >60 cells per condition. Statistical significance was determined using one-way ANOVA. *P = 0.0498 for VPS35[D620N] ± MLi-2. Scale bars, 10 μm. (**B**) LRRK2 inhibition restores ciliogenesis in VPS35[D620N] MEFs. Indicated MEF cells were serum-starved for 24 hours ± 200 nM MLi-2 or DMSO. Cilia and nuclei were visualized with anti-Arl13b antibody or DAPI, respectively; ciliation was determined by direct counting. Error bars represent SEM from three independent experiments by two different people 3 years apart, where >200 cells per condition were scored. Statistical significance was determined using one-way ANOVA. ***P = 0.0004 for VPS35[D620N] + DMSO versus VPS35[D620N] + MLi-2. Scale bars, 10 μm.

Most pRab10 is localized adjacent to the mother centriole in the perinuclear region, and it is retained there by interaction with pRab effectors that couple to motor proteins (*13**,* *50*). The aberrant accumulation of pRabs on stressed and/or damaged lysosomes leads to the recruitment of JIP3/JIP4 and RILPL1, which would be predicted to relocalize lysosomes to the perinuclear region in an LRRK2-dependent manner. We thus evaluated the perinuclear distribution of lysosomes in VPS35[D620N] MEFs, with and without LRRK2 activity, using LAMP1 as a lysosome marker. As shown in Fig. 4A, MLi-2 treatment did not alter the distribution of LAMP1-positive lysosomes in WT MEFs. However, MLi-2 treatment decreased the perinuclear concentration of lysosomes in VPS35[D620N] MEFs. These data support the conclusion that a low level of lysosome-associated pRab proteins affects lysosomal localization in VPS35[D620N] MEFs.

### MLi-2 treatment restores ciliogenesis in VPS35[D620N] MEFs

We showed previously that LRRK2 activity causes a 42% decrease in ciliation in LRRK2[R1441G] MEFs: Cells that are normally 60% ciliated are only 35% ciliated in the absence of MLi-2 [figure 6C in (*13*)]. As shown in Fig. 4B, VPS35[D620N] MEFs also showed decreased ciliation, consistent with their high content of pRab10: 43% of VPS35[D620N] MEFs were ciliated compared with 63% upon MLi-2 treatment. In this case, despite having twice the level of pRab10 as LRRK2[R1441C] MEFs [figure 3C in (*39*)], ciliation has only decreased 32%. This difference may be explained by decreased levels of RILPL1 in VPS35[D620N] MEFs, as our previous work has shown that the LRRK2-mediated ciliation blockade depends on both pRab10 and its RILPL1 binding partner (*13*).

### LRRK2 activity drives association of RILPL1 with TMEM55B, a lysosomal integral membrane protein

**Fig. 5.**
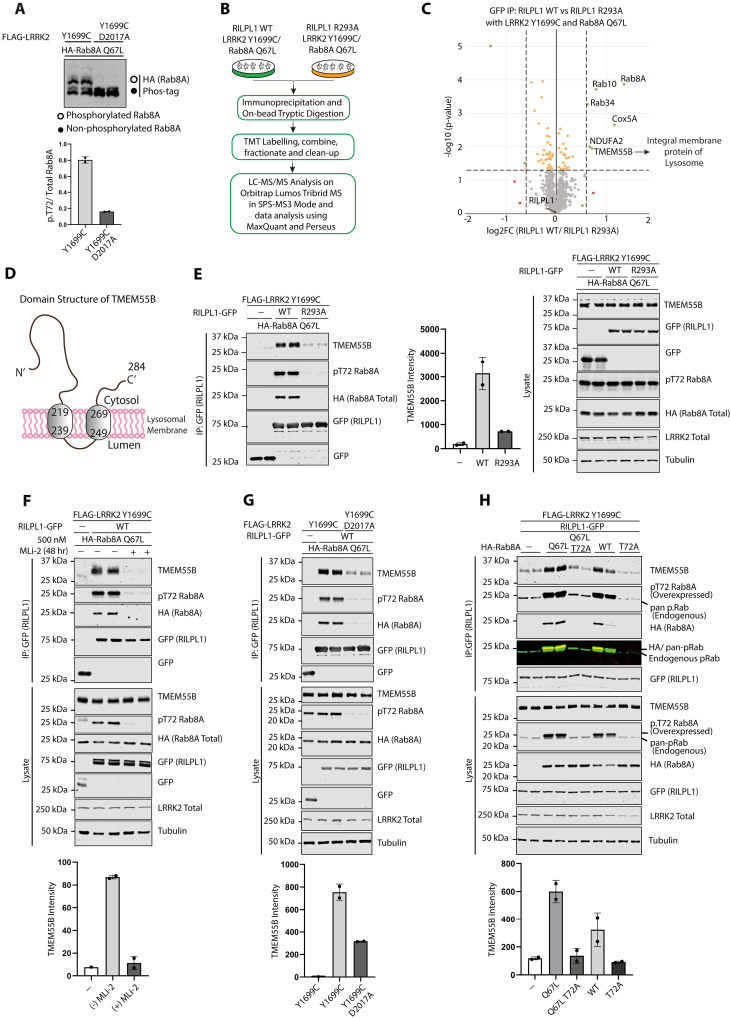
Association of RILPL1 with a lysosomal membrane integral protein, TMEM55B, mediated by LRRK2 activity. (**A**) HEK293 cells were transiently transfected with HA-Rab8A[Q67L] (GTP-bound mutant) either in the presence of LRRK2[Y1699C] (kinase active mutant) or LRRK2[D2017A] (kinase inactive mutant). Twenty-four hours after transfection, 20 μg of WCL was analyzed on a Phos-tag gel and immunoblot was developed using the Bio-Rad ChemiDoc Imaging System. Each lane represents cell extracts obtained from a different dish of cells. (**B**) Depiction of the workflow for the TMT label–based MS analysis of GFP IPs from HEK293 cells transiently transfected as in (A) with RILPL1-GFP WT or RILPL1-GFP [R293A] (non-pRab8/10 binding mutant). (**C**) Volcano plot depicting the fold enrichment of proteins between IPs from RILPL1-GFP WT and RILPL1-GFP R293A mutant (*P* value adjusted by two-tailed Student’s *t* test, which is corrected by permutation-based FDR of 5%; Curtain link: https://curtain.proteo.info/#/422e26ba-1120-42cd-be4f-caeb115aeea1) (table S6). (**D**) TMEM55B domain structure. (**E** to **H**) Transfections and immunoprecipitation of the indicated proteins were performed as in (B) and analyzed by quantitative immunoblot analysis using the LI-COR Odyssey CLx Western blot imaging system and indicated antibodies. Quantitation of immunoblotting data (performed using ImageStudioLite software version 5.2.5, RRID:SCR_013715) is shown as mean ± SEM.

We hypothesized that in VPS35[D620N] cells, if LRRK2 was recruited to lysosomes and phosphorylated Rab proteins at this location, this could trigger recruitment of RILPL1 to the lysosome, where RILPL1 might interact with other lysosomal protein(s). To explore this further, we undertook a RILPL1 enrichment MS analysis in human embryonic kidney (HEK) 293 cells overexpressing LRRK2[Y1699C] together with the GTP locked form of Rab8A (Rab8A[Q67L]). As control, we used a RILPL1 mutant in which the critical Arg^293^ residue required for binding LRRK2 phosphorylated Rab8A is mutated to Ala; this would block RILPL1 from being recruited to the lysosome by binding to phosphorylated Rab8A (*11*). Control phos-tag immunoblot analysis (*51*) confirmed that in these experiments, Rab8A is phosphorylated by LRRK2 to a ~50% stoichiometry maximizing the opportunity to identify downstream targets (Fig. 5A). Using an isobaric tandem mass tag (TMT) affinity enrichment MS workflow (Fig. 5B), we compared interactors of WT RILPL1 with mutant RILPL1[R293A] (Fig. 5C and table S6). As expected, WT RILPL1 is associated with Rab8A (fig. S8A) and Rab10 (fig. S8B) to a greater extent than the RILPL1[R293A] mutant (Fig. 5C). In addition, four other proteins also interacted more strongly with WT RILPL1, namely, TMEM55B (Fig. 5C and fig. S8C), a lysosomal integral membrane protein (*52*), Rab34 (Fig. 5C and fig. S8D) (not a known LRRK2 substrate), and two mitochondrial proteins: NDUFA2 (Fig. 5C and fig. S8E) and COX5A (Fig. 5C and fig. S8F).

We focused on TMEM55B, which is composed of 284 residues and 2 transmembrane domains with N- and C-terminal domains facing the cytosol (Fig. 5D). Immunoblotting experiments validated the MS studies, confirming that endogenous TMEM55B coimmunoprecipitated with WT but not mutant RILPL1[R293A] coexpressed with LRRK2[Y1699C] (Fig. 5E). Furthermore, treatment with MLi-2 (Fig. 5F), or introduction of a mutation that ablates LRRK2 kinase activity (D2017A) (Fig. 5G), markedly inhibited association of RILPL1 with TMEM55B. GTP-locked Rab8A[Q67L] associated with TMEM55B to a moderately greater extent than WT Rab8A (Fig. 5H), consistent with previous data showing that Rab proteins in the GTP-bound conformation interact with higher affinity with RILPL1 (*11*). Together, these results suggest that when LRRK2 is recruited to VPS35[D620N] lysosomes, it phosphorylates Rab proteins, which recruit RILPL1 to the lysosome, thereby inducing its interaction with TMEM55B.

### LRRK2 activity promotes colocalization of RILPL1 and TMEM55B at the lysosome

**Fig. 6.**
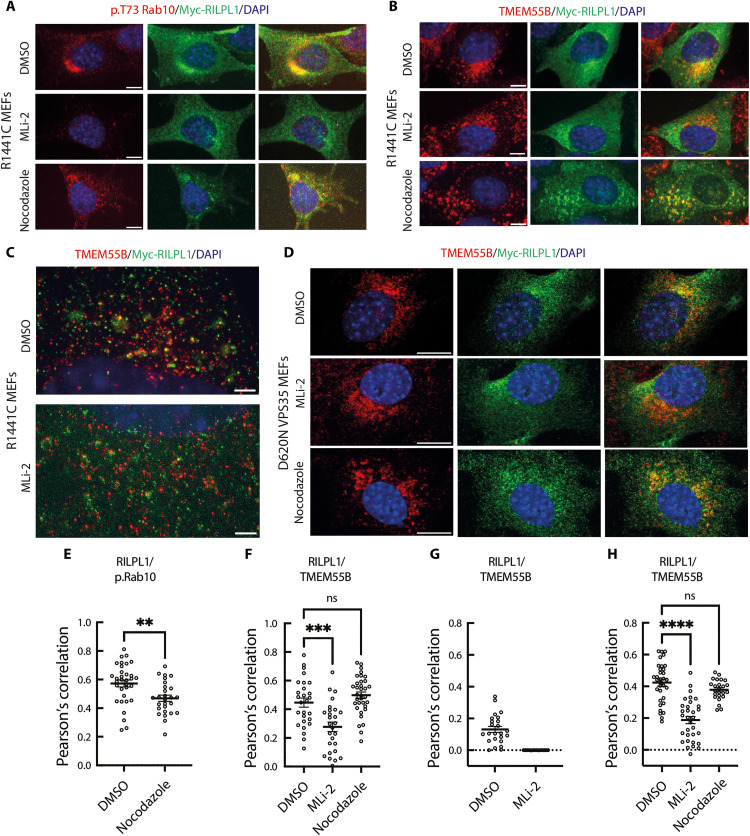
LRRK2-dependent colocalization of RILPL1 and TMEM55B in LRRK2[R1441C] and VPS35[D620N] MEF cells. Immunofluorescence microscopy of cells expressing transfected Myc-RILPL1 and stained using anti-Myc antibody and antibodies to detect endogenous p.Rab10 or TMEM55B. (**A**) R1441C MEFs treated with or without 200 nM MLi-2 for 2 hours or 20 μM nocodazole for 2 hours to depolymerize microtubules. Red, p.Rab10; green, Myc- RILPL1. Merged images are shown at the far right in this and subsequent panels. (**B**) R1441C MEFs stained to detect endogenous TMEM55B and transfected Myc-RILPL1 as in (A). (**C**) R1441C MEFs visualized using expansion microscopy to detect endogenous TMEM55B (red) and transfected Myc-RILPL1 (green). (**D**) VPS35[D620N] MEFs stained with antibodies to detect endogenous TMEM55B (red) or transfected Myc-RILPL1 (green), and merged image. Scale bars, 10 μm (A to D). (**E** to **H**) Pearson’s correlation coefficients between RILPL1 and p.Rab10 or TMEM55B as indicated from experiments presented in (A) to (D). Significance was determined using ordinary one-way ANOVA *P* < 0.001 for multiple comparisons or *t* test for paired comparisons. (E) **P = 0.0032; (F) ***P = 0.0003; (H) ****P < 0.0001. (E), (F), and (H) represent two independent replicates with 25 to 35 cells total analyzed. (G) represents one replicate with 15 to 24 cells analyzed.

Using confocal microscopy, we investigated the localization of endogenous pRab10, overexpressed Myc-RILPL1, and endogenous TMEM55B in LRRK2[R1441C] MEFs. As shown previously (*13**,* *14*), we observed substantial colocalization of pRab10 and RILPL1 in the perinuclear region that was partially affected by nocodazole treatment that disrupts microtubule dynamics (Fig. 6, A and E). Treatment with MLi-2 abolished pRab10 phosphorylation without markedly affecting RILPL1 localization. Some colocalization of Myc-RILPL1 with endogenous TMEM55B was observed in the perinuclear region, and this was also substantially reduced with MLi-2 treatment but not nocodazole (Fig. 6, B and F). In cells, membrane-bound organelles rely on the microtubule-based cytoskeleton to achieve their subcellular distributions. When microtubules are depolymerized with nocodazole, organelles are released and diffuse throughout the cytoplasm. Under these conditions, two proteins on the same membrane compartment will diffuse together and should retain any “true” colocalization.

To observe the lysosomes in more detail, we used expansion microscopy, revealing that a low percentage of lysosomes determined by endogenous TMEM55B expression colocalized with Myc-RILPL1 (Fig. 6, C and G). Treatment with MLi-2 greatly reduced any colocalization of endogenous TMEM55B and Myc-RILPL1 (Fig. 6, C and G). Similar results were obtained in VPS35[D620N] MEFs treated with MLi-2, but not nocodazole, reducing the interaction of RILPL1 with TMEM55B (Fig. 6, D and H). These data support the finding that the LRRK2 pathway is driving the association of RILPL1 with TMEM55B on a low proportion of lysosomes.

### C terminus of RILPL1 interacts with N terminus of TMEM55B

**Fig. 7.**
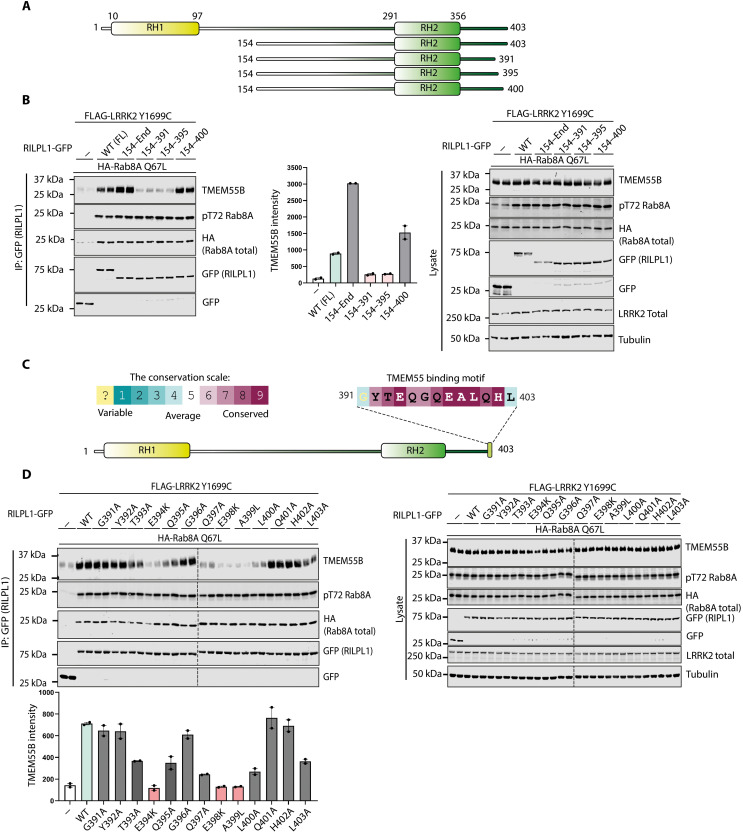
RILPL1 interacts with TMEM55B via a conserved motif at the C terminus. (**A**) Domain structure of full-length and truncated mutants of RILPL1 used in this study. (**B**) HEK293 cells were transiently transfected with the indicated proteins and lysed 24 hours after transfection. GFP-RILPL1 immunoprecipitations (top) or cell extracts (bottom) were subjected to quantitative immunoblot analysis using the LI-COR Odyssey CLx Western blot imaging system and indicated antibodies. Quantitation of immunoblotting data (performed using ImageStudioLite software version 5.2.5, RRID:SCR_013715) is shown as mean ± SEM. (**C**) Analysis of C-terminal residue conservation of RILPL1 using the ConSurf motif software (RRID: SCR_002320) (*81*) and conservation score scale. (**D**) As in (B).

Truncation mutagenesis revealed the removal of the last eight amino acids of RILPL1 (Fig. 7, A and B, and fig. S9, A and B), which are evolutionarily conserved (Fig. 7C), abolished binding to TMEM55B. Mutation of several residues within this motif (Glu^394^Lys, Glu^398^Lys, and Ala^399^Leu) markedly suppressed the interaction of RILPL1 with TMEM55B (Fig. 7D). We have termed this region the TMEM55-binding motif, which is not conserved in RILP and RILPL2. Consistent with this, neither RILP nor RILPL2 coimmunoprecipitates with TMEM55B (fig. S9, C and D).

**Fig. 8.**
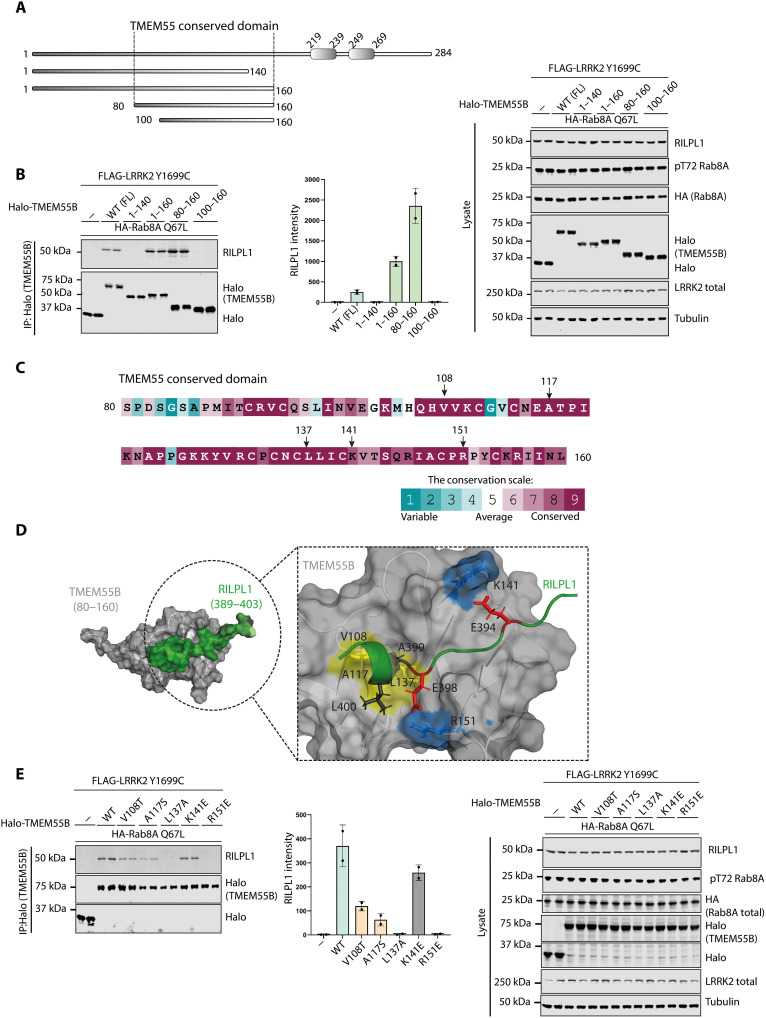
N-terminal conserved domain of TMEM55B facilitates the binding of RILPL1. (**A**) Domain structure of full-length and truncated mutants of TMEM55B used here. (**B**) HEK293 cells were transiently transfected with the indicated proteins and lysed 24 hours after transfection. Halo-TMEM55B immunoprecipitations (top) or cell extracts (bottom) were subjected to quantitative immunoblot analysis using the LI-COR Odyssey CLx Western blot imaging system and indicated antibodies. Quantitation of immunoblotting data (performed using ImageStudioLite software version 5.2.5, RRID:SCR_013715) is shown as mean ± SEM. (**C**) Analysis of C-terminal residue conservation of TMEM55B using the ConSurf motif software (*81*) and conservation score scale. (**D**) Key residues that are involved in the interaction between TMEM55B and RILPL1 were analyzed by AlphaFold2 model of TMEM55B (80 to 160) and RIPL1 (389 to 403). (**E**) As in (B).

Mutagenesis analysis revealed that the minimum region of TMEM55B required for interaction with RILPL1 spans an evolutionary conserved region encompassing residues 80 to 160 (Fig. 8, A to C, and fig. S10, A and B) that we have termed the TMEM55-conserved domain. AlphaFold (*53*) predicts that this region adopts a globular fold having a hydrophobic groove along one surface aligned with conserved residues (Fig. 8D). Using AlphaFold2.ipynb ColabFold notebook with AlphaFold2-multimer-v2 and AMBER structure relaxation, we modeled how full-length RILPL1 would interact with full-length TMEM55B (fig. S10C). The top models predicted (fig. S10D) an interaction between the RILPL1 TMEM-binding motif and the hydrophobic groove on the surface of TMEM-conserved domain, with two additional electrostatic interactions involving conserved residues R151 and K141 (Fig. 8D). Mutational analysis revealed that the R151E but not the K141E mutation ablated binding of TMEM55B to RILPL1 (Fig. 8E). Mutation of the conserved hydrophobic groove residues (V108T, A117S, and L137A) also reduced RILPL1 binding (Fig. 8E).

Cells express another TMEM55 isoform termed TMEM55A, which is highly related to TMEM55B, with 55% identity in sequence and 86% identity in sequence within the TMEM55-conserved domain (fig. S11A). Overexpression studies reveal that TMEM55A, although expressed at ~2-fold lower levels than TMEM55B, also interacted with RILPL1 (fig. S11B).

### Knockdown of TMEM55B increases cellular level of RILPL1 without affecting Rab phosphorylation

**Fig. 9.**
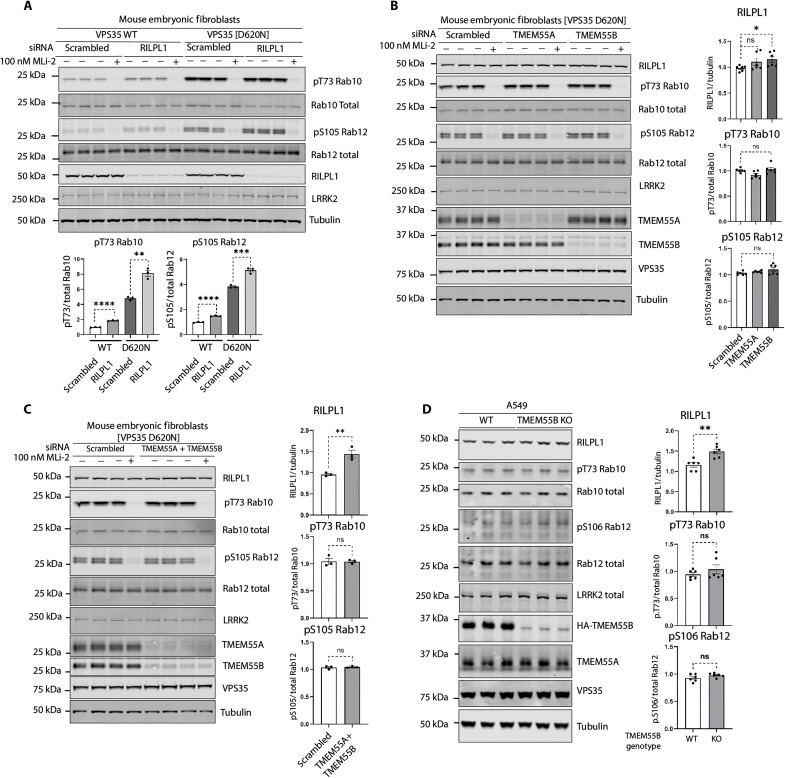
Effect of knockdown or knockout of RILPL1, TMEM55A, and TMEM55B. (**A** to **C**) VPS35[D620N] homozygous KI MEFs were transfected with the indicated siRNA for 72 hours, and 100 nM MLi-2 [or DMSO, 0.1% (v/v)] was added 1 hour before lysis. Cell lysates were analyzed by quantitative immunoblot analysis using the LI-COR Odyssey CLx Western blot imaging system and the indicated antibodies. Quantitation of immunoblotting data (performed using ImageStudioLite software version 5.2.5, RRID:SCR_013715) is shown as mean ± SEM. Data were analyzed using two-tailed unpaired *t* test (**P < 0.01, ***P < 0.001, and ****P < 0.001). (**D**) *TMEM55B* CRISPR KO A549 cells and KO cells complemented with virally transduced *3HAxTMEM55B* (WT) were lysed and analyzed as in (A).

We next studied the effect that small interfering RNA (siRNA) knockdown of RILPL1 had on LRRK2 activity and found that this moderately increased LRRK2-mediated phosphorylation of Rab10 and Rab12 in WT and VPS35[D620N] MEFs (Fig. 9A). We next observed that siRNA knockdown of TMEM55B, but not TMEM55A, in VPS35[D620N] MEFs increased cellular levels of RILPL1, consistent with the notion that RILPL1 binding to TMEM55B accelerated the degradation of RILPL1 (Fig. 9B). Knockdown of TMEM55A or TMEM55B or both did not affect LRRK2-mediated phosphorylation of Rab10 or Rab12 in VPS35[D620N] MEFs (Fig. 9, B and C). We also generated CRISPR *TMEM55B* knockout A549 cells and also observed an increase in RILPL1 levels in WCL without affecting Rab10 or Rab12 phosphorylation (Fig. 9D).

### The lysosomotropic agent LLOMe also induced recruitment of RILPL1 to the lysosome

**Fig. 10.**
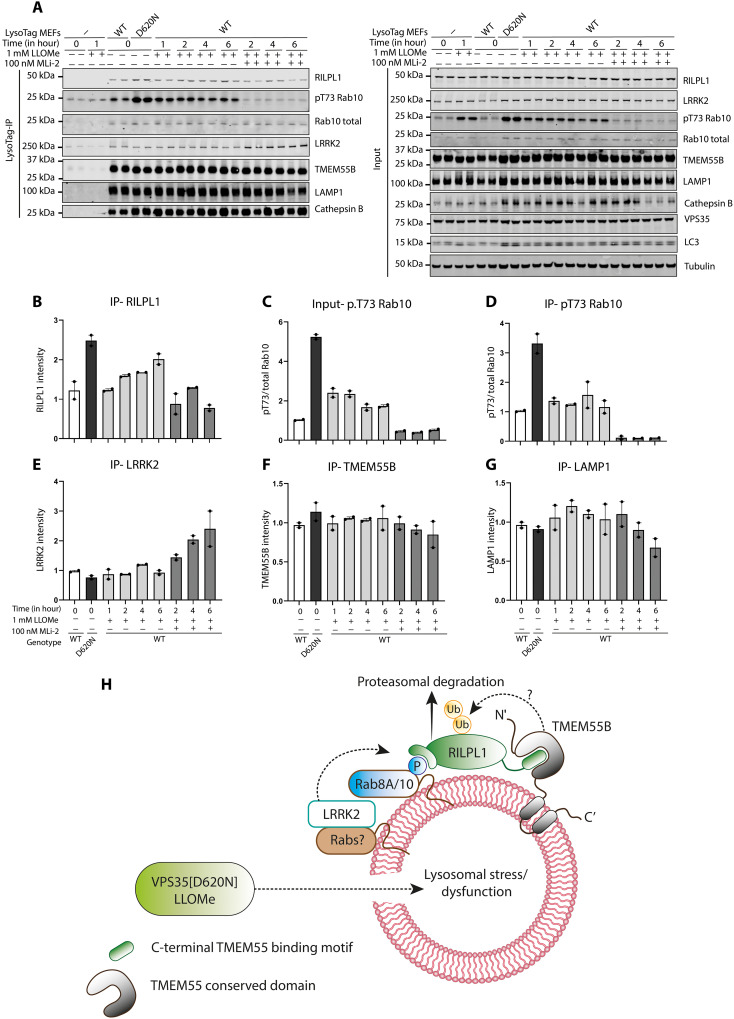
Effect of LLOMe compared to D620N mutation on recruitment of RILPL1 to the lysosome (**A** to **G**) WT or VPS35[D620N] homozygous KI MEFs expressing ± LysoTag (TMEM192-3xHA) were treated as indicated ± 1 mM LLOMe and ± 100 nM MLi-2 for the indicated time points. Cells were homogenized, and lysosomes were immunoprecipitated. Four micrograms of WCL or lysosome extract was analyzed by quantitative immunoblot analysis using the LI-COR Odyssey CLx Western blot imaging system and indicated antibodies. Quantitation of immunoblotting data (performed using ImageStudioLite software version 5.2.5, RRID:SCR_013715) is shown as mean ± SEM. (**H**) Model of how lysosomal dysfunction resulting from VPS35[D620N] mutation recruits and activates LRRK2 to the lysosome, resulting in phosphorylation of Rab proteins, which in turn triggers the recruitment of RILPL1 and its binding to TMEM55A/B.

Last, we treated WT MEFs with the lysosomotropic agent LLOMe at 1 mM for 1 to 6 hours to determine whether this resulted in the recruitment of RILPL1 to the lysosome. These experiments revealed that LLOMe induced a 1.5-fold increase in the levels of RILPL1 at the lysosome at later 2- to 6-hour time points (Fig. 10, A and B). In VPS35[D620N] MEFs, LLOMe treatment enhanced lysosomal RILPL1 levels more, namely, ~2.5-fold (Fig. 10, A and B). LLOMe induced a moderate increase in pRab10/total Rab10 levels in cell lysates (Fig. 10C) and lysosomes (Fig. 10D) that was also ~2-fold lower than levels caused by the D620N mutation. As observed in Fig. 2A, MLi-2 induced a moderate increase in the association of LRRK2 to the lysosome, but this was not observed in the absence of MLi-2 with LLOMe treatment (Fig. 10E).

## DISCUSSION

The retromer complex controls retrograde sorting of cargos from the endosome back to the trans-Golgi network, as well as recycling cargo from the endosome to the cell surface. The D620N mutation affects the core VPS35 backbone subunit of the retromer complex and has been suggested to disrupt the retromer’s retrograde cargo trafficking pathway (*54**–**56*). Structural analysis of the retromer complex has indicated that the D620N mutation might affect oligomerization of the complex, but this has not been definitely established (*57*). IP MS studies indicate that the D620N mutation moderately impaired interaction with FAM21, a key member of the WASH complex that binds to VPS35, and this mutation thus impairs association of the WASH complex to the endosomes (*54**,* *55*). How VPS35[D620N] mutations stimulate LRRK2 pathway activity is not known, and there is no strong evidence that LRRK2 and the retromer complex interact directly.

Our preferred model that is supported by our data is that the D620N mutation, by disrupting the retromer’s retrograde cargo trafficking pathway, ultimately leads to a specific form of lysosomal dysfunction, and this is what triggers LRRK2 recruitment to the lysosome and induces an enhanced phosphorylation of Rab proteins such as Rab10 at that location. Many previous studies have pointed toward lysosomal damage/dysfunction triggering LRRK2 pathway activation. For example, treatment of cells with lysosome-damaging agents such as chloroquine (*58**,* *59*) or LLOMe (*12*) triggers LRRK2 recruitment to the lysosome, inducing its activation and enhanced phosphorylation of Rab8A and Rab10 on the lysosome. Chloroquine was also reported to induce relocalization of Rab8/Rab10 effectors EHBP1 and EHBP1L1 to the lysosome (*58*), whereas LLOMe induced lysosomal recruitment of the pRab10 motor adapter protein JIP4 (*12*) as well as the RAB7 GTPase-activating protein TBC1D15 (*60*). Similarly in macrophages, pathogen infection induces lysosomal damage and was shown to result in relocalization of LRRK2 to the damaged lysosome where it phosphorylated Rab8A, and this recruited the ESCRT-III component CHMP4B that orchestrates the repair of lysosome damage (*31*). A recent study has shown that overexpression of LRRK2 in 293A cells induces perinuclear clustering of lysosomes that display elevated phosphorylation of Rab12 and RILPL1 that are consistent with our findings in this study (*49*).

None of the above studies used VPS35[D620N] as a possible form of more physiological lysosome stress that is relevant to PD. We pursued our D620N data for the levels of all of these proteins in WT and D620N cell lysates and LysoTag IP (fig. S12). The data show that for CHMP4B (fig. S12A), TBC1D15 (fig. S12B), EHBP1 (fig. S12C), and JIP3 (fig. S12D) levels are moderately decreased in cell lysates of D620N mutation compared to WT, but there is no clear-cut enrichment of any of these proteins to the lysosome. Treatment of D620N cells with 100 nM MLi-2 for 48 hours did not alter the levels of these proteins, suggesting that reduced expression in the D620N cells is not related to LRRK2 kinase activity. More work will be required to understand why the D620N mutation moderately reduces the levels of these proteins. For JIP4 (fig. S12E), there were no significant changes or enrichment to the lysosome. We did not detect RILP and RILPL2 in our datasets.

Our data reveal that the VPS35[D620N] mutation in MEFs is having a significant impact on the lysosomal enrichment of proteins. We observed that the lysosomal abundance of ~150 proteins was altered over twofold in VPS35[D620N] MEFs compared to WT (Fig. 1D). A recent study also reported profound protein abundance changes in LysoTag IP fractions of H4 neuroglioma VPS35 KO cells (*61*). Notably, treatment of D620N MEFs with MLi-2 only reduced lysosomal association of a single protein notably, namely, RILPL1. Our data also suggest that LLOMe treatment of MEF VPS35 WT cells induces recruitment of RILPL1 to the lysosome, albeit to a lower extent than observed with the D620N mutation (Fig. 10, A and B).

We also find that in homozygous VPS35[D620N] MEFs as well as mouse brain and lung, the levels of RILPL1 in whole-cell extracts are reduced compared to WT and this is largely reversed by treatment with MLi-2. Our model is that once RILPL1 is recruited to the lysosome in VPS35[D620N] cells and tissues, this leads to its accelerated degradation, thereby lowering the steady-state level in WCLs. Our data suggest that this degradation is mediated by the proteasome pathway, and in future work, it would be interesting to explore whether reduction of RILPL1 levels in WCLs is a useful biomarker for monitoring lysosomal dysfunction that is of relevance to PD.

Our data indicate that the recruitment of RILPL1 to TMEM55B is dependent on LRRK2 kinase activity, as this is blocked by both MLi-2 and a mutation that ablates the ability of RILPL1 to bind phospho-Rab proteins. In VPS35[D620N] cells, our data demonstrate that LRRK2 is recruited to the damaged/dysfunctional lysosome by a yet unknown mechanism. Using overexpression and immunofluorescence, others have demonstrated recruitment of LRRK2 to lysosomes following chloroquine and LLOMe treatment. Consistent with this, we observed increased recruitment of green fluorescent protein (GFP)–LRRK2 to the lysosome in VPS35[D620N] MEFs compared to WT (Fig. 1F). It should be noted that it is hard to observe recruitment of LRRK2 to the lysosome in these LysoTag IP experiments with WT and VPS35[D620N] cells in the absence of MLi-2 (Fig. 1C). It is possible that during the cell homogenization and/or the washing steps that are involved in the LysoTag IP steps, endogenous LRRK2 may dissociate from the lysosome as it may not be strongly anchored to the organelle. However, in the presence of MLi-2 that traps LRRK2 in the active conformation, we are able to see enhanced localization of LRRK2 to the lysosome in the LysoTag IP (Fig. 2C). It is possible that LRRK2 locked in the active conformation when complexed with LRRK2 associates more stably with the lysosome. One mechanism by which LRRK2 could be recruited to the lysosome involves Rab binding to LRRK2’s N-terminal ARM domain. At the lysosome, LRRK2 would phosphorylate Rab proteins including Rab8A and Rab10 either unusually on the lysosome or on adjacent vesicles. This in turn recruits RILPL1 to the lysosome where it then binds to the integral lysosomal transmembrane receptor protein TMEM55B. Our imaging data in Fig. 6C suggest that only a low proportion of lysosomes have RILPL1 associated with TMEM55B. It is possible that these represent the actively stressed lysosomes that are being targeted by the LRRK2 pathway. Microscopy studies confirm that MLi-2 treatment ablates the binding of RILPL1 and TMEM55B. A model of how binding of RILPL1 and TMEM55 may be regulated is shown in Fig. 10H.

The binding mechanism that we have defined for how RILPL1 interacts with TMEM55B involves evolutionary conserved residues on both proteins. Our data suggest that TMEM55A could also be involved in binding to RILPL1 (fig. S11B). The copy number levels of TMEM55A and TMEM55B in HEK293 cells are 30,000 (TMEM55A) and 141,000 (TMEM55B); in MEFs, 23,000 (TMEM55A) and 87,000 (TMEM55B); and in mouse brain, 58,000 (TMEM55A) and 37,000 (TMEM55B) (https://copica.proteo.info/#/home). Previous work suggested that TMEM55A and TMEM55B might act as phosphatidylinositol-4,5-bisphosphate 4-phosphatases based on the presence of a CX_5_R motif that is located within the TMEM55-conserved domain (*62*). The AlphaFold model of this domain bears no resemblance to any inositol phosphatase that we are aware of, and it seems likely that this domain functions as a receptor protein to recruit specific proteins such as RILPL1 to the lysosome. Consistent with this, another recent study has reported that recombinant TMEM55B lacked detectable inositol phosphatase activity when expressed in vitro (*52*).

Previous work has shown that TMEM55B controls lysosomal movement in cells by binding to the JIP4 motor adaptor protein and thereby linking the lysosome to a dynein-dependent microtubule transport machinery (*52*). It was also reported that TMEM55B levels are transcriptionally up-regulated following TFEB and TFE3 activation by starvation or cholesterol-induced lysosomal stress and that this pathway could coordinate lysosome movement in response to stress conditions (*52*). Sequence analysis indicates that JIP4 has a conserved motif (residues 887 to 898) that is similar in sequence to the C-terminal TMEM binding motif on RILPL1. In future work, it would be important to explore whether JIP4 interacts with TMEM55B via this motif and how these binding interactions might be regulated. Further work is also required to establish how the D620N retromer–induced lysosomal stress or LLOMe affects TMEM55B. We have not observed an increase in TMEM55A (fig. S12F) and TMEM55B (fig. S12G) levels in WT and D620N MEFs or D620N MEFs treated ± MLi-2. TMEM55B has been reported to be regulated by phosphorylation (*63*) and binding with components of the mammalian target of rapamycin complex 1 (mTORC1) pathway (*64*). In future work, it would be important to understand how TMEM55B is regulated and whether it plays a role in sensing lysosomal dysfunction and what the signaling pathways modulate this process. If TMEM55B and components that it regulates are controlled by lysosomal stress, this could point toward the development of biomarkers to detect lysosomal stress pathways of relevance to PD.

## MATERIALS AND METHODS

### Plasmids

All plasmids used here were obtained from the MRC Protein Phosphorylation and Ubiquitylation Unit (PPU) Reagents and Services and are available to request via the MRC PPU Reagents and Services website (https://mrcppureagents.dundee.ac.uk). These are listed in Supplementary Materials and Methods.

### Antibodies

All antibodies used in this study are listed in Supplementary Materials and Methods.

### Generation of MEFs and stable expression of LysoTag and GolgiTag

WT and homozygous VPS35[D620N] knock-in MEFs were isolated from littermate-matched mouse embryos at day E12.5, resulting from crosses between heterozygous mice (The Jackson Laboratory strain no.: 023409; RRID:IMSR_JAX:023409) using a protocol described previously (*65*). Genotypes were verified via allelic sequencing.

WT and homozygous VPS35[D620N] knock-in MEFs stably expressing the LysoTag (*43*) or GolgiTag (*47*) were generated using the protocol described in (*66*). Briefly, littermate-matched primary MEFs were first immortalized by SV40 large T antigen viral transduction before the stable expression of target proteins [LysoTag, GolgiTag, or HA-Empty (vector only encoding a HA tag)] by a subsequent viral transduction. To generate desired viruses, HEK293 FRT cells were seeded to give a 60% confluency for transfection the following day. A mixture of 12 μg of DNA (6 μg of target DNA, 3.8 μg of GAG/POL, and 2.2 μg of vesicular stomatitis virus glycoprotein) and 36 μl of polyethylenimine (PEI; Polysciences) transfection reagent was diluted to 500 μl in Opti-MEM reduced serum medium (Gibco) and incubated for 30 min before adding to cells dropwise. The transfection-medium mixture was discarded after 24 hours, and 10 ml of fresh medium was added. The next day, the medium was harvested and filtered through a 0.45-μm filter before storing at −80°C.

MEF cells were plated in 10-cm dishes to give a 70% confluency the following day for viral transduction by the addition of 5 ml of virus with 5 ml of fresh medium and polybrene (10 μg/ml; Sigma-Aldrich, TR-1003-G). After 24-hour incubation, the viral medium was discarded. Cells were given time to recover if required. Cells were selected for viral uptake by the addition of a selection agent until nontransduced control plates died. For SV40 immortalization of MEFs, 200 μM hygromycin (InvivoGen, ant-hg-5) was used for selection pressure. For organelle tag introduction to MEFs, puromycin (2 μg/ml) was used for selection pressure.

### Generation of A549 *TMEM55B* KO cells by CRISPR-Cas9 and introduction of 3xHA TMEM55B into A549 *TMEM55B* KO cells

A full transcript map of the TMEM55B locus was constructed by combining data from both National Center for Biotechnology Information (NC_000014.9) and Ensembl (ENSG00000165782). KO guide RNAs were selected to target exon 2 to ensure complete disruption of all possible transcripts. Three sets of CRISPR-Cas9 guide RNAs were designed to target exon 2 of TMEM55B: a pair targeting exon 2 (sense A and antisense A); G1, a single-guide RNA (3′-GCCCTTAACTAGCCCGGACAG-5′); and G2, a single-guide RNA (3′-GACTCGGCAGGTGATCATAG-5′). A549 cells were cotransfected with 1 μg of each plasmid and 2 μg of PEI mixture supplemented with Opti-MEM. Twenty-four hours after transfection, cells were kept in Dulbecco’s modified Eagle’s medium (DMEM) containing puromycin (2 μg/ml) for 48 hours. After the recovery, cell pools were analyzed for the depletion of TMEM55B expression by immunoblotting, and afterwards, single cells were sorted using fluorescence-activated cell sorting. Following 2 to 3 weeks of recovery, promising clones were verified by polymerase chain reaction (PCR), shotgun cloning, and sequencing. To rescue the expression of TMEM55B in A549 (RRID: CVCL_LI35) *TMEM55B* KO cells (RRILD: CVCL_D3VW), we used a retrovirus approach to introduce 3xHA-TMEM55B as described previously (*67*).

### Cell culture, transfection, treatments, and lysis

WT and homozygous VPS35[D620N] knock-in MEF cells isolated from littermate-matched mouse embryos were cultured in DMEM (Gibco) containing 10% (v/v) fetal bovine serum (FBS; Sigma-Aldrich), 2 mM l-glutamine, penicillin (100 U/ml), and streptomycin (100 μg/ml) supplemented with 1× nonessential amino acid solution and 1 mM sodium pyruvate (Life Technologies, Gibco). HEK293 (RRID: CVCL_0045) cells were purchased from the American Type Culture Collection and maintained in DMEM containing 10% (v/v) FBS, 2 mM l-glutamine, penicillin (100 U/ml), and streptomycin (100 μg/ml). All cells were grown at 37°C temperature with 5% CO_2_ in a humidified atmosphere and regularly tested for mycoplasma contamination. Transient transfections were performed in HEK293 cells 24 hours before cell lysis using PEI at around 60 to 70% confluency. Transfections for coimmunoprecipitation assays were done in 10-cm cell culture dishes using 3 μg of Flag-LRRK2 Y1699C or Flag-LRRK2 Y1699C D2017A as indicated, 1 μg of HA control or HA-Rab8A Q67L, and 2 μg of RILPL1-GFP or Halo-TMEM55B cDNA construct per dish diluted in 1 ml of Opti-MEM and 20 μg of PEI mixture and incubated for 30 min before being added to the medium. HEK293 cells were treated with 500 nM MLi-2 inhibitor before the transfections. VPS35[D620N] MEFs were treated with 100 nM MLi-2 after 24 hours of seeding either for 48 hours or at different time points (4, 8, 12, and 24 hours). VPS35[D620N] MEFs were treated with either cycloheximide (50 μg/ml) alone, cycloheximide (50 μg/ml) + 10 μM MG-132, or cycloheximide (50 μg/ml) + lysosomal protease inhibitor cocktails (5 μM E64D, 10 μM leupeptin, and 10 μM pepstatin A) after 24 hours of seeding either for 8 or 12 hours. Unless otherwise stated, cells were lysed in an ice-cold lysis buffer containing 50 mM Tris-HCl (pH 7.5), 1% (v/v) NP-40 alternative or 1% (v/v) Triton X-100, 10% (v/v) glycerol, 150 mM NaCl, 1 mM sodium orthovanadate, 50 mM sodium fluoride, 10 mM sodium β-glycerophosphate, 5 mM sodium pyrophosphate, microcystin-LR (0.1 μg/ml), and one tablet of cOmplete Mini (EDTA-free) protease inhibitor (Merck, 11836170001). Protein lysates were clarified by centrifugation at 17,000*g* for 10 min and quantified by Bradford assay. Human biological cells (A549/HEK293) were sourced ethically, and their research use was in accord with the terms of the informed consent under an institutional review board–approved protocol. Detailed methods for cell transfection and cell lysis were described previously (*68**,* *69*).

### Immunofluorescence microscopy

MEF cells were grown in a six-well plate seeded at 50 to 60% confluency in 2 ml of DMEM (Gibco) containing 10% (v/v) FBS (Sigma-Aldrich), 2 mM l-glutamine, penicillin (100 U/ml), and streptomycin (100 μg/ml). After 24 hours, cells were transfected with 2 μg of plasmid DNA per well in Fugene (3:1 ratio) according to the manufacturer’s instructions. After another 24 hours, cells were trypsinized and plated onto 12-mm glass coverslips (Fisher Scientific, USA) in a six-well plate (Fisher Scientific, USA) at 60% confluency. After another 24 hours, cells were treated with MLi-2 or nocodazole before processing for microscopy according to this method (*70*). For expansion microscopy, details can be found at (*71*).

All images were obtained using a spinning disk confocal microscope (Yokogawa) with an electron-multiplying charge-coupled device camera (Andor, UK) and a 63× oil immersion objective or a Zeiss LSM 900 microscope acquired using Zen 3.4 and a 63× objective. Images were converted to maximum intensity projections using Fiji (https://fiji.sc/) (RRID: SCR_002285). For quantitation using CellProfiler (RRID:SCR_007358; http://cellprofiler.org), image maximum intensity projections were processed in batch for using this macro (*72*). Pearson’s correlation coefficients were obtained as described in (*73*). Quantification of perinuclear lysosome using LAMP1 as marker was described in (*74*). For colocalization of LRRK2 and TMEM192, 4 μg of GFP-LRRK2 plasmid was electroporated in LysoTag VPS35 WT and D620N MEFs, 30,000 to 40,000 suspended cells per 10 μl using the following electroporation pulse parameters (poring pulse parameters—voltage: 200 V, length: 5 ms, interval: 50 ms, number of cycles: 2, decay rate: 10%, polarity: + and transfer pulse parameters—voltage: 20 V, length: 5 ms, interval: 50 ms, number of cycles: 5, decay rate: 40%, polarity: +/−). After this, cells were transferred to the six-well plates with coverslips and kept for at least 18 hours in a 37°C incubator. The coverslips were washed with phosphate-buffered saline (PBS) and fixed using 4% paraformaldehyde and processed for immunostaining using anti-HA (for TMEM192) and anti-GFP antibody (for LRRK2).

### siRNA-mediated knockdown of target proteins in MEFs

For siRNA knockdown of proteins of interest, ON-TARGETplus Mouse Tmem55A siRNA-SMARTpool (catalog no. L-059670-01-0005), ON-TARGETplus Mouse Tmem55B siRNA-SMARTpool (catalog no. L-047594-01-0005), ON-TARGETplus Mouse Rilpl1 siRNA-SMARTpool (catalog no. L-063225-01-0005), and ON-TARGETplus nontargeting pool (#D-001810-10-05) were purchased from Horizon Discovery Ltd. MEF cells were seeded in a six-well format at 200,000 cells per well for transfection the following day (at 60 to 70% confluency). Cells were transfected using Lipofectamine RNAiMAX Transfection Reagent (Invitrogen, catalog no. 13778075) according to the manufacturer’s protocol (https://assets.thermofisher.com/TFS-Assets/LSG/manuals/Lipofectamine_RNAiMAX_Reag_protocol.pdf). Briefly, 50 pmol of siRNA was diluted in 150 μl of Opti-MEM and combined with 10 μl of Lipofectamine RNAiMAX in 150 μl of Opti-MEM per well. The two mixtures were incubated together at room temperature for 5 min, and 250 μl was added dropwise to cells, which were harvested 72 hours after transfection.

### Mouse models and MLi-2 diet study

All animal studies were ethically reviewed and carried out in accordance with the Animals (Scientific Procedures) Act 1986 and regulations set by the University of Dundee and the U.K. Home Office, and the GSK Policy on the Care, Welfare and Treatment of Animals. Animal studies and breeding were approved by the University of Dundee ethical committee and performed under a U.K. Home Office project licence. Mice were housed at an ambient temperature (20° to 24°C) and humidity (45 to 55%) and were maintained on a 12-hour light/12-hour dark cycle, with free access to food and water. VPS35[D620N] knock-in mice (Jax strain no.: 023409; RRID:IMSR_JAX:023409) crossed with LysoTag knock-in mice (Jax strain no.: 035401; RRID:IMSR_JAX:035401) were used for this study. Mouse genotyping was performed by PCR using genomic DNA isolated from tail clips or ear biopsies.

For the experiment shown in Fig. 3 (D and E), littermate or age-matched male and female VPS35[D620N] homozygous knock-in mice at 4 months of age were used. Mice were allowed to acclimatize to the control rodent diet (Research Diets D01060501, Research Diets, New Brunswick, NJ) for 14 days before being placed on study. On day 1 of the study, one group (six mice) received modified rodent diet (Research Diets D01060501) containing MLi-2 and formulated by Research Diets to provide a concentration of 60 mg/kg per day on the basis of an average food intake of 5 g/day for 14 days; the other group (12 mice) received untreated diet (Research Diets D01060501) for 14 days and served as the control group. The dose of MLi-2 and the length of the in-diet treatment used for this study were based on (*48*). Bodyweight and food intake were assessed twice weekly. On the last day of the study, all mice were euthanized by cervical dislocation, and the brain and lung tissues were transferred to ice-cold PBS and processed immediately as described below.

### Mouse tissue lysis for organelle IP

The brain and lung tissue were transferred to a cold room and subjected to homogenization using a 2-ml Dounce homogenizer (VWR, Tissue grinders, Potter-Elvehjem type, 432-0206) by 25 strokes in 1 ml of KPBS [136 mM KCl and 10 mM KH_2_PO_4_ (pH 7.2) using KOH] supplemented with 1× cOmplete Mini (EDTA-free) protease inhibitor (Merck, 11836170001) and 1× PhosSTOP phosphatase inhibitor (Merck, 4906837001). The homogenate was collected and precleared by centrifugation at 1000*g* for 2 min at 4°C. The precleared homogenate was collected and lysed in a 1:1 dilution with an ice-cold lysis buffer as stated above except the detergent used was 1% Triton X-100 (v/v) instead of 1% NP-40 (v/v). The lysate was kept on ice for 10 min before clarification by centrifugation at 17,000*g* for 10 min at 4°C followed by protein concentration estimation and immunoblot analysis as stated above. A detailed method for isolation of organelles from mouse tissues is described in (*75*). Isolated organelles were processed as described in (*75*).

### Organelle isolation from MEFs

Lysosomes were isolated from WT and homozygous VPS35[D620N] knock-in MEF cells stably expressing the LysoTag (TMEM192-3xHA) via HA IP. MEFs cultured to confluency in 15-cm cell culture dishes were washed briefly with PBS before cell scraping into 1 ml of KPBS buffer. Cells were pelleted at 1500*g* for 2 min at 4°C. The KPBS supernatant was then aspirated, and 1 ml of fresh KPBS was used to resuspend the cell pellet. For downstream WCL analysis, 50 μl of this cell suspension was retained and lysed in 1% Triton X-100 (v/v) lysis buffer as described above. The remaining cell suspension (950 μl) was subjected to ball-bearing homogenization with an isobiotec cell homogenizer with 10-μm clearance, involving 10 passes back and forth of the sample through the ball-bearing device. The homogenized cell sample was recovered and centrifuged (1500*g* for 2 min at 4°C). The supernatant was then applied to 100 μl of anti-HA Pierce magnetic beads (Thermo Fisher Scientific) in a 1.5-ml Eppendorf, mixing gently via pipetting up and down five times. The sample tube was then placed on an IBI Scientific belly dance orbital shaker, set to full speed, at 4°C for 5 min. Following the 5-min immunoprecipitation incubation, IP tubes were placed on a magnetic tube holder for 30 s, before the supernatant was removed. The magnetic beads, with bound lysosomes, were then washed three times in 1 ml of KPBS buffer (transferring the beads to a fresh tube at the third wash), using the magnetic sample holder to draw beads from solution during each wash. After the final KPBS wash, the bead sample was either lysed directly in 1% Triton X-100 lysis buffer for immunoblotting analysis or stored dry at −80°C before sample preparation for proteomic analysis. A detailed method for organelle isolation and analysis is described in (*76*).

### Coimmunoprecipitation assays

GFP or Halo IP was performed according to the manufacturer’s protocol (for GFP IP: GFP-Trap Agarose—ChromoTek GmbH, for Halo IP: https://promega.co.uk/-/media/files/resources/protocols/technical-manuals/0/halolink-resin-protocol.pdf) as described in (*77*). Briefly, lysates were incubated with either GFP-Trap agarose beads (Chromotek) or HaloLink Resin (Promega) for 1 to 2 hours (20 μl of packed resin/1 mg of lysate). Immunoprecipitates were washed three times with wash buffer [50 mM tris-HCl (pH 7.5) and 150 mM NaCl] and eluted by adding 2× NuPAGE LDS sample buffer. The mixture was then incubated at 95°C for 10 min, and the eluent was collected by centrifugation through a 0.22-μm Spin-X column (CLS8161, Sigma-Aldrich). Eluted samples were supplemented with 1% (by volume) β-mercaptoethanol and denatured at 70°C for 10 min before being subjected to immunoblot analysis.

### Quantitative immunoblotting analysis

Quantitative immunoblotting analysis was performed according to the protocol described in (*77*). Briefly, 10 to 20 μg of lysate or 25% of the immunoprecipitated samples were loaded onto NuPAGE 4 to 12% Bis-Tris Midi Gels (Thermo Fisher Scientific, catalog no. WG1402BOX or WG1403BOX) or self-cast 10% bis-tris gels [0.375 M bis-tris (pH 6.8), 10% (w/v) acrylamide, 1% (v/v) tetramethylethylenediamine (TEMED), and 0.05% (w/v) ammonium persulfate (APS)] and electrophoresed at 130 V for 2 hours with NuPAGE Mops SDS running buffer (Thermo Fisher Scientific, catalog no. NP0001-02). At the end of electrophoresis, proteins were electrophoretically transferred onto a nitrocellulose membrane (GE Healthcare, Amersham Protran Supported 0.45 μm NC) at 90 V for 90 min on ice in transfer buffer [48 mM tris and 39 mM glycine supplemented with 20% (v/v) methanol]. The membranes were blocked with 5% (w/v) skimmed milk powder dissolved in TBS-T [50 mM tris base, 150 mM sodium chloride (NaCl), and 0.1% (v/v) Tween 20] at room temperature for 1 hour. Membranes were washed three times with TBS-T and incubated in primary antibody overnight at 4°C*.* Before secondary antibody incubation, membranes were washed three times for 15 min each with TBS-T. The membranes were incubated with secondary antibody for 1 hour at room temperature. Thereafter, membranes were washed with TBS-T three times with a 15-min incubation for each wash, protein bands were acquired via near-infrared fluorescent detection using an Odyssey CLx imaging system, and intensities of bands were quantified using Image Studio Lite (version 5.2.5, RRID:SCR_013715).

For Phos-tag analysis, samples were supplemented with 10 mM MnCl_2_ before loading them onto the gel. Phos-tag gel consisted of stacking gel [4% (w/v) acrylamide, 0.125 M tris-HCl (pH 6.8), 0.2% (v/v) TEMED, and 0.08% (w/v) (APS)] and resolving gel [10% (w/v) acrylamide, 375 mM tris-HCl (pH 8.8), 75 μM Phos-tag reagent (MRC PPU Reagents and Services), 150 μM MnCl_2_, 1% (v/v) TEMED, and 0.05% (w/v) APS]. Samples were loaded onto the gel after centrifugation at 17,000*g* for 1 min and electrophoresed at 90 V with running buffer [25 mM tris-HCl, 192 mM glycine, and 0.1% (w/v) SDS]. For immunoblot analysis, gels were washed three times for 10 min with 48 mM tris-HCl, 39 mM glycine, 10 mM EDTA, and 0.05% (w/v) SDS followed by one wash with 48 mM tris-HCl, 39 mM glycine, and 0.05% (w/v) SDS for 10 min. Proteins were then transferred onto the nitrocellulose membranes at 100 V for 180 min on ice using transfer buffer as mentioned before. Membranes were blocked with 5% (w/v) skimmed milk dissolved in TBS-T at room temperature. Next, the membranes were incubated with the primary antibodies overnight at 4°C. After washing the membrane with TBS-T (three times for 10 min), membranes were incubated with horseradish peroxidase–conjugated secondary antibody diluted in 5% skimmed milk in TBS-T at room temperature for 1 hour. After washing the membranes in TBS-T (five times for 10 min), protein bands were developed using a ChemiDoc system (Bio-Rad) after adding the enhanced chemiluminescence reagent (SuperSignal West Dura Extended Duration, Thermo Fisher Scientific) to the membranes.

### Sample preparation, labeling, fractionation, LC-MS/MS, and data analysis for TMT experiments

The washed GFP IP beads were dissolved in a 100-μl buffer containing 2 M urea, 50 mM tris-HCl (pH 7.5), and 1 mM dithiothreitol incubated on a thermomixer at 32°C for 30 min and then supplemented with final 20 mM iodoacetamide (IAA) for another 30 min in the dark. Sequencing-grade trypsin (250 ng) was added to the samples and incubated on a thermomixer at 1200 rpm agitation for 2 hours, the supernatant was transferred to new 15-ml Eppendorf tubes, and the tryptic digestion was continued for 12 hours. The reaction was quenched by adding final 1% (v/v) trifluoroacetic acid (TFA), and peptides were purified using in-house prepared strong cation exchange stage tips. Eluted peptides were vacuum-dried, and TMT labeling was performed (11-plex TMT, Thermo Fisher Scientific) by following the manufacturer’s instructions. After labeling verification, samples were pooled to equal volumes and vacuum-dried. To improve the coverage, pooled TMT-labeled mix was subjected to mini-basic reversed-phase liquid chromatography (LC) fractionation as described in (*78*) and generated a total of four fractions, which are vacuum-dried and stored at −80°C until LC-MS/MS analysis.

Each fraction was analyzed on a Thermo Orbitrap Lumos Tribrid mass spectrometer in a Data-Dependent Acquisition (DDA) MS3 mode. The peptides were loaded on a 2-cm precolumn and resolved on a 50-cm analytical column at a flow rate of 300 nl/min. The full scan was acquired at 120,000 mass/charge ratio (*m*/*z*) resolution in the mass range of 375 to 1500 *m*/*z* and measured using an Orbitrap mass analyzer. The top 10 data-dependent MS2 scans were isolated by setting quadrupole mass filter at 0.7 Da and fragmented using 35% collisional-induced dissociation. The fragment ions were measured using ion trap in a rapid scan mode. Synchronous precursor selection (MS3) for top 10 fragment ions in the mass range of 400 to 1200 *m*/*z* was isolated and fragmented using 65% higher energy collisional dissociation and measured at 50,000 *m*/*z* 200 resolution using an Orbitrap mass analyzer. The automatic gain control (AGC) targets were set at 2 × 10^5^, 2 × 10^4^, and 5 × 10^4^ for MS1, MS2, and MS3 scans, respectively, with ion injection times set at 50 ms for MS1 and MS2 and 120 ms for MS3 scans. The raw MS data were processed using MaxQuant software suite (*79*) (RRID:SCR_014485, version 1.6.6.0; https://maxquant.org/). The data type was set as a reporter ion MS3. The data were searched against Human UniProt (https://www.uniprot.org/, release: 2017_3) by selecting the default contaminants. Carbamidomethylation of Cys was used as a static modification, and oxidation (M), acetyl (protein N-term), deamidation (NQ), and phosphorylation (STY) were set as variable modifications. One percent false discovery rate (FDR) was applied at Peptide Spectral Match (PSM) and protein levels. The protein group.txt files were then further processed using Perseus software suite (*80*) (RRID:SCR_015753, version 1.6.0.15; https://maxquant.org/perseus/) for statistical analysis. A detailed protocol can be found at (*77*).

### Sample preparation, LC-MS/MS, and data analysis for DIA experiments

To reduce the lysates, 5 mM tris(2-carboxyethyl)phosphine was used. The samples were placed on a thermomixer (Eppendorf, UK) at 60°C with 1100 rpm for 30 min. After cooling to room temperature, 20 mM IAA was added for alkylation. During alkylation, the samples were shielded from light and placed on a thermomixer at 25°C with 1100 rpm for 30 min. Each sample was then mixed with 5% (v/v) SDS and 1.2% (v/v) phosphoric acid and further diluted with 6× wash buffer [90% MeOH and 10% Triethylammonium bicarbonate (TEABC) at pH 7.2]. The samples were thoroughly vortexed and loaded onto S-Trap (ProtiFi, USA) columns by centrifugation at 1000*g* for 1 min, and the flow-through collected from the columns was discarded. After sample loading, the S-Trap columns were washed three times with 150 μl of wash buffer. On-column digestion was performed by incubating 60 μl (1.5 μg) of trypsin/Lys-C mix (MS grade, Promega, UK) in 50 mM TEABC solution at pH 8 on the samples on a thermomixer at 47°C for 1 hour before reducing the incubation temperature to 22°C for overnight digestion. The samples were then eluted into 1.5-ml low-binding tubes (Eppendorf, UK) by centrifugation with 60 μl of 50 mM TEABC solution at pH 8, followed by 60 μl of 0.15% (v/v) formic acid (FA) aqueous solution, and then 60 μl of elution buffer [80% acetonitrile (ACN) with 0.15% FA in aqueous solution] twice. The eluted samples were immediately snap-frozen on dry ice and dried at 35°C using the SpeedVac Vacuum Concentrator (Thermo Fisher Scientific, UK). The dried samples were resuspended in 60 μl of solution containing 3% (v/v) ACN and 0.1% (v/v) FA and further incubated on a thermomixer at 22°C with 1200 rpm for 30 min followed by 30-min sonication in a water bath. The sample concentration was then estimated using a NanoDrop instrument (Thermo Fisher Scientific, UK) by measuring the solution absorbance at 224-nm wavelength.

LC-MS/MS was performed using an UltiMate 3000 RSLC nano–high-performance liquid chromatography (HPLC) system (Thermo Fisher Scientific, UK) coupled to an Orbitrap Exploris 480 mass spectrometer (Thermo Fisher Scientific, UK). Four micrograms of each sample was loaded onto the nano-HPLC system individually. Peptides were trapped by a precolumn (Acclaim PepMap 100, C18, 100 μm × 2 cm, 5 μm, 100 Å) using an aqueous solution containing 0.1% (v/v) TFA. The peptides were then separated by an analytical column (PepMap RSLC C18, 75 μm × 50 cm, 2 μm, 100 Å) at 45°C using a linear gradient of 8 to 25% solvent B (an 80% ACN and 0.1% FA solution) for 98 min, 25 to 37% solvent B for 15 min, 37 to 95% solvent B for 2 min, 95% solvent B for 8.5 min, 95 to 3% solvent B for 0.5 min, and 3% solvent B for 9.5 min. The flow rate was set at 250 nl/min for all experiments. Data were acquired in DIA mode containing 45 isolated mass/charge ratio windows ranging from 350 to 1500. Collision-induced dissociation with nitrogen gas was used for peptide fragmentation.

The DIA MS experiment’s raw data were analyzed using the DIA-NN software (RRID: SCR_022865, version 1.8) (*44*) using a library-free search mode. Trypsin/P was selected as the digestive enzyme, and up to two missed cleavages were allowed. Carbamidomethylation at cysteine residue was set as a fixed modification, while oxidation at methionine residue was included as a variable modification. The software automatically detected and adjusted the mass error (parts per million). A protein identification cutoff of 1% FDR was used, and a protein quantification required a minimum of two peptides in five of six samples. The search results were then imported into Perseus software (*80*) (RRID:SCR_015753, version 1.6.0.15; https://maxquant.org/perseus/) for statistical analysis. For the LysoTag IP samples, IP samples were first compared against the relevant mock IP samples to classify proteins substantially enriched at the lysosome, using a fold change of >1.5 and *P* value of <0.05 (fig. S1). The lysosomal enriched proteins were then compared against genotypes or treatments to investigate protein level changes at the lysosome organelle. For the WCL samples, proteins were directly compared against genotypes or treatments to determine the proteome changes in the cells. Significant up-/down-regulated proteins (fold change > |1.5| and *P* < 0.05) obtained from LysoTag IP and WCL lysate samples of MEF VPS35 WT versus D620N were then submitted to metascape (RRID:SCR_016620, version 5.3) (*46*) for enrichment analysis. Enrichment of GO biological processes pathway, GO molecular functions, and GO cellular components with *P* < 0.01 were reported in fig. S3. The text files generated from Perseus software were then imported into an in-house software, Curtain 2.0 (RRID: SCR_024465), for data visualization. A detailed protocol can be found at (*66*).

### Statistical analysis

All statistical analyses were performed in GraphPad Prism (RRID:SCR_002798, version 9.3.1; http://graphpad.com/). Two-tailed unpaired *t* test was performed for statistical comparison of two groups. One-way analysis of variance (ANOVA) was performed for statistical comparison of three or more groups.

## Supplementary Material

20231213-1

## Supplementary Materials

### This PDF file includes:

Tables S1 to S3 Legends for tables S4 to S6 Figs. S1 to S12 References

### Other Supplementary Material for this manuscript includes the following:

Tables S4 to S6

Erratum published 27 March 2026:
The original version of the Research Article contained errors in Fig. 5 and table S6. The following corrections have been implemented: • The originally published table S6 erroneously conflated data from two distinct tables in the IP-MS dataset. Table S6 has been corrected to include only data from one of the two distinct tables. • The placement of RILPL1 in Fig. 5C has been corrected based on the data from the corrected table S6. • The Curtain data link in the caption for Fig. 5C has been corrected from “https://curtain.proteo.info/#/4f15d6c8-9192-4bb2-a2ff-da7aa542025c” to “https://curtain.proteo.info/#/422e26ba-1120-42cd-be4f-caeb115aeea1.” Figure 5 and table S6 have been corrected and the Supplementary Materials PDF and the main text PDF and HTML have been updated. The original version of the Supplementary Materials PDF is available here:
