# Supplementary material for: Parkinson’s VPS35[D620N] mutation induces LRRK2-mediated lysosomal association of RILPL1 and TMEM55B
Source: Sci Adv. 2023 Dec 13;9(50):eadj1205. doi: 10.1126/sciadv.adj1205 (PMC10848721; doi:10.1126/sciadv.adj1205)
Supplement: Supplementary file 4 — Erratum published 27 March 2026: The original version of the Research Article contained errors in Fig. 5 and table S6. The following corrections have been implemented: • The originally published table S6 erroneously conflated data from two distinct tables in the IP-MS dataset. Table S6 has been corrected to include only data from one of the two distinct tables. • The placement of RILPL1 in Fig. 5C has been corrected based on the data from the corrected table S6. • The Curtain data link in the caption for Fig. 5C has been corrected from “https://curtain.proteo.info/#/4f15d6c8-9192-4bb2-a2ff-da7aa542025c” to “https://curtain.proteo.info/#/422e26ba-1120-42cd-be4f-caeb115aeea1.” Figure 5 and table S6 have been corrected and the Supplementary Materials PDF and the main text PDF and HTML have been updated. The original version of the Supplementary Materials PDF is available here: [file sciadv.adj1205_sm.v1.pdf]

Science Advances
AAAS

Supplementary Materials for

Parkinson's VPS35[D620N] mutation induces LRRK2-mediated lysosomal association of RILPL1 and TMEM55B

Prosenjit Pal *et al.*

Corresponding author: Prosenjit Pal, p.z.pal@dundee.ac.uk; Dario R. Alessi, d.r.alessi@dundee.ac.uk

Sci. Adv. **9**, eadj1205 (2023)
DOI: 10.1126/sciadv.adj1205

The PDF file includes:

Tables S1 to S3
Legends for tables S4 to S6
Figs. S1 to S12
References

Other Supplementary Material for this manuscript includes the following:

Tables S4 to S6

Supplementary Materials and Methods

Table S1. Plasmids

All plasmids used in this study were obtained from the MRC PPU Reagents and Services are available to request via the MRC PPU Reagents and Services website (https://mrcppureagents.dundee.ac.uk).

**DU number**	Plasmid
**DU13156**	pCDNA5 FRT/TO *GFP* Empty
**DU72471**	pCDNA5 FRT/TO *Halo* Empty
**DU49303**	pCMV5 *HA* Empty
**DU13363**	pCDNA5 FRT/TO *GFP-LRRK2*
**DU26486**	pCMV5 *FLAG-LRRK2* Y1699C
**DU52703**	pCMV5 *FLAG-LRRK2* Y1699C D2017A
**DU51184**	pCDNA5 FRT/TO *HA-Rab8A*
**DU52575**	pCDNA5 FRT/TO *HA-Rab8A* T72A
**DU51181**	pCDNA5 FRT/TO *HA-Rab8A* Q67L
**DU68868**	pCDNA5 FRT/TO *HA-Rab8A* Q67L T72A
**DU27305**	pCDNA5 FRT/TO *RILPL1-GFP*
**DU68072**	pCDNA5 FRT/TO *RILPL1-GFP* R293A
**DU72537**	pCDNA5 FRT/TO *RILPL1-GFP* G391A
**DU72538**	pCDNA5 FRT/TO *RILPL1-GFP* Y392A
**DU72539**	pCDNA5 FRT/TO *RILPL1-GFP* T393A
**DU71243**	pCDNA5 FRT/TO *RILPL1-GFP* E394K
**DU72540**	pCDNA5 FRT/TO *RILPL1-GFP* Q395A
**DU72541**	pCDNA5 FRT/TO *RILPL1-GFP* G396A
**DU72542**	pCDNA5 FRT/TO *RILPL1-GFP* Q397A
**DU71241**	pCDNA5 FRT/TO*RILPL1-GFP* E398K

**DU71238**	pCDNA5 FRT/TO *RILPL1-GFP* A399L
**DU71235**	pCDNA5 FRT/TO *RILPL1-GFP* L400A
**DU72543**	pCDNA5 FRT/TO *RILPL1-GFP* Q401A
**DU72461**	pCDNA5 FRT/TO *RILPL1-GFP* H402A
**DU72544**	pCDNA5 FRT/TO *RILPL1-GFP* L403A
**DU72259**	pCDNA5 FRT/TO *RILPL1-GFP* 154-End
**DU61811**	pCDNA5 FRT/TO *RILPL1-GFP* 154-391
**DU71249**	pCDNA5 FRT/TO *RILPL1-GFP* 154-395
**DU71250**	pCDNA5 FRT/TO *RILPL1-GFP* 154-400
**DU70318**	pCDNA5 FRT/TO *Halo-TMEM55B*
**DU72563**	pCDNA5 FRT/TO *Halo-TMEM55B* V108T
**DU72565**	pCDNA5 FRT/TO *Halo-TMEM55B* A117S
**DU72532**	pCDNA5 FRT/TO *Halo-TMEM55B* L137A
**DU72375**	pCDNA5 FRT/TO *Halo-TMEM55B* K141E
**DU72569**	pCDNA5 FRT/TO *Halo-TMEM55B* R151E
**DU72129**	pCDNA5 FRT/TO *Halo-TMEM55B* 1-140
**DU68815**	pCDNA5 FRT/TO *Halo-TMEM55B* 1-160
**DU72132**	pCDNA5 FRT/TO *Halo-TMEM55B* 80-160
**DU72015**	pCDNA5 FRT/TO *Halo-TMEM55B* 100-160
**DU70216**	pCMV *VSVG*
**DU40867**	pBabed *SV40* Large T Antigen
**DU70217**	pCMV *GAG/POL*
**DU68534**	pLJC5 *TMEM115-3xHA* (GolgiTag)
**DU68356**	pLJC5 *TMEM192-3xHA* (LysoTag)
**DU70022**	pLJC5 *HA-Empty*
**DU77348**	pLJC5 *3xHA TMEM55B*

**DU69593**	Sense A Guide RNA for *TMEM55B* CRISPR-Cas9 KO cell generation
**DU69594**	Anti-sense A Guide RNA for *TMEM55B* CRISPR-Cas9 KO cell generation
**DU69595**	G1 Single Guide RNA for *TMEM55B* CRISPR-Cas9 KO cell generation
**DU69596**	G2 Single Guide RNA for *TMEM55B* CRISPR-Cas9 KO cell generation

Table S2. Primary Antibodies

Antibody Target	Source	Catalogue Number (RRID)	Dilution
GM130	Abcam	ab52649 (AB_880266)	1:1000
LAMP1	Cell Signaling Technology	3243 (AB_2134478)	1:1000
TMEM55B/ PIP4P1	Proteintech	23992-1-AP (AB_2879391)	1:1000 (IB)<br>1:500 (IF)
JIP4/ SPAG9	Cell Signaling Technology	5519 (AB_10828724)	1:1000
ACBD3	Sigma-Aldrich	HPA015594 (AB_1844491)	1:1000
Cathepsin B	Cell Signaling Technology	31718 (AB_2687580)	1:1000
α-Tubulin	Cell Signaling Technology	3873 (AB_1904178)	1:10000
Halo	Promega	G9211 (AB_2688011)	1:1000
GAPDH	Santa Cruz Biotechnology	Sc-32233 (AB_627679)	1:10000
phospho-S935 LRRK2	MRC PPU Reagents and Services, UoD	UDD2 10(12) (AB_2921228)	1 µg/ml
LRRK2 Total C-terminal	Neuromab	N241A/34 (AB_2877351)	1 µg/ml

Phospho-Rab8A (Pan-Thr-specific)	Abcam	ab230260 (AB_2814988)	1:1000
HA	Roche	3F10 (AB_2314622)	1:1000
GFP	Cell Signaling Technology	2956 (AB_1196615)	1:1000
Rab10 Total	Nanotools	0680-100/Rab10-605B11 (AB_2921226)	1 µg/ml
phospho-T73 Rab10	Abcam	ab230261 (AB_2811274)	1:1000
Rab12	MRC PPU Reagents and Services, University of Dundee	SA227 (AB_2921227)	1 µg/ml
phospho-S106 Rab12	Abcam	ab256487 (AB_2884880)	1:1000
VPS35	StressMarq	SMC-602 (AB_2820301)	1:5000
LC3A/B	Cell Signaling Technology	4108 (AB_2137703)	1:1000
RILPL1	Abcam	Ab302492 (AB_2936945)	1:1000
TMEM55A	MRC PPU Reagents and Services, University of Dundee	DA241 (AB_2936451)	1 µg/ml
Myc	BioLegend	626802 (AB_2148451)	1:250 (IF)

Table S3. Secondary Antibodies

Antibody Target	Company	Catalogue number (RRID)	Dilution
IRDye 800CW Goat anti-Rabbit IgG	LI-COR	926-32211 (AB_621843)	1:25,000
IRDye 680CW Goat anti-Mouse IgG	LI-COR	926-68070 (AB_10956588)	1:25,000
IRDye 680CW Goat anti-Rat IgG	LI-COR	926-68074 (AB_10956736)	1:25,000
IRDye 800CW Donkey anti-Rabbit IgG	LI-COR	926-32213 (AB_621848)	1:25,000
IRDye 680LT Donkey anti-Mouse IgG	LI-COR	926–68 022 (RRID:AB_10715072)	1:25,000
IRDye 680LT Donkey anti-Goat IgG	LI-COR	926–68 024 (RRID:AB_10706168)	1:25,000
Donkey anti-Rabbit 568 Alexa Fluor	Thermo Fisher	A-10042 (RRID:AB_2534017)	1:500 (IF)
Donkey anti-Mouse 488 Alexa Fluor	Thermo Fisher	A-21202 (RRID:AB_141607)	1:500 (IF)

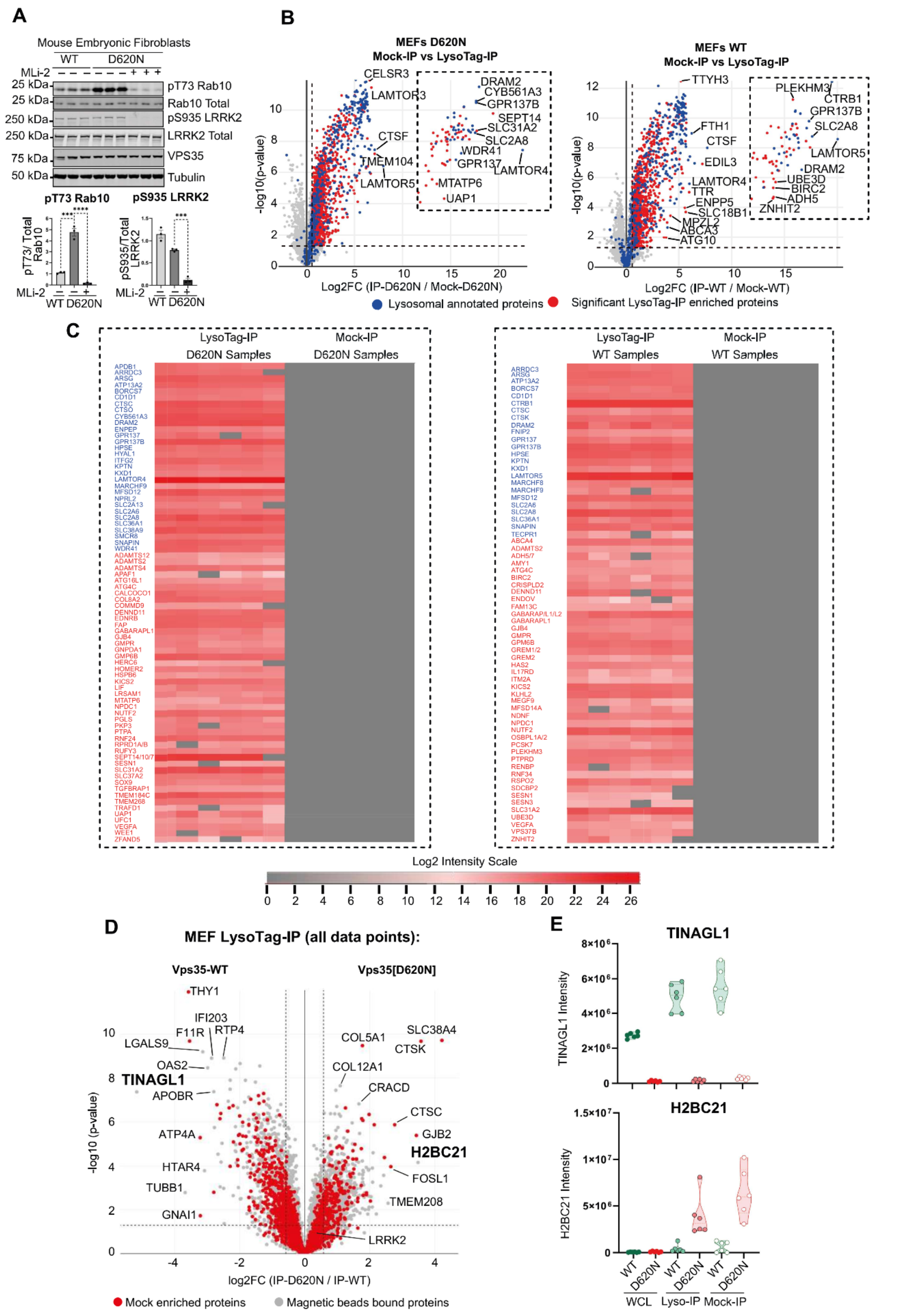

Fig. S1. Further Mass Spectrometry analysis of wild type and D620N MEFs. (A) Littermate-matched wild type (WT) and *VPS35[D620N]* homozygous knock-in (KI) MEFs were treated ± 100 nM MLi-2 for 2 h prior to lysis. Lysate were subjected to quantitative immunoblot analysis using the LI-COR Odyssey CLx Western Blot imaging and the indicated antibodies. Technical replicates represent cell extract obtained from different dishes of cells. Quantitation of immunoblotting data (performed using ImageStudioLite software version 5.2.5, RRID:SCR_013715) is shown as mean ± SEM. Data were analyzed using two-tailed unpaired student's *t*-test (*** p< 0.001, **** p< 0.001). (B) The volcano plot show the result of MEFs VPS35[D620N] not

expressing LysoTag (Mock-IP) versus VPS35[D620N] LysoTag-IP (TMEM-192-3xHA) samples (left) (Curtain link: https://curtain.proteo.info/#/e7b85272-37e8-4785-9879-954f19c96784) and MEFs VPS35-WT not expressing LysoTag (Mock-IP) versus VPS35-WT LysoTag-IP samples (right) (Curtain link: https://curtain.proteo.info/#/4399d86e-67d2-40f6-af3c-6e237c54a589) (Table S4). The blue dots represent lysosomal proteins annotated in the GO terms database (GO:0005764), while the red dots represent significantly enriched proteins with fold change > 1.5 and p-value < 0.05. The black dotted line box indicates proteins confidently detected in the LysoTag-IP samples but not in the Mock-IP samples. (C) The log2 MS intensities of these proteins are shown. (D) The volcano plot (Curtain link: https://curtain.proteo.info/#/18c40d8a-f678-4d7e-976a-f70c85683ccc) shows the proteome changes of all detected proteins in MEFs VPS35-WT versus VPS35[D620N] LysoTag-IP experiments. The red dots represent mock-enriched proteins with fold change > 1.5 and p-value < 0.05, as determined in the experiments shown in Supplementary Figure 1A. The gray dots represent proteins bound to magnetic beads, which do not show significant enrichment. (D) The raw MS intensities of two non-specific proteins that associate with the magnetic beads (TINAGL1 and H2BC21) were shown for reference.

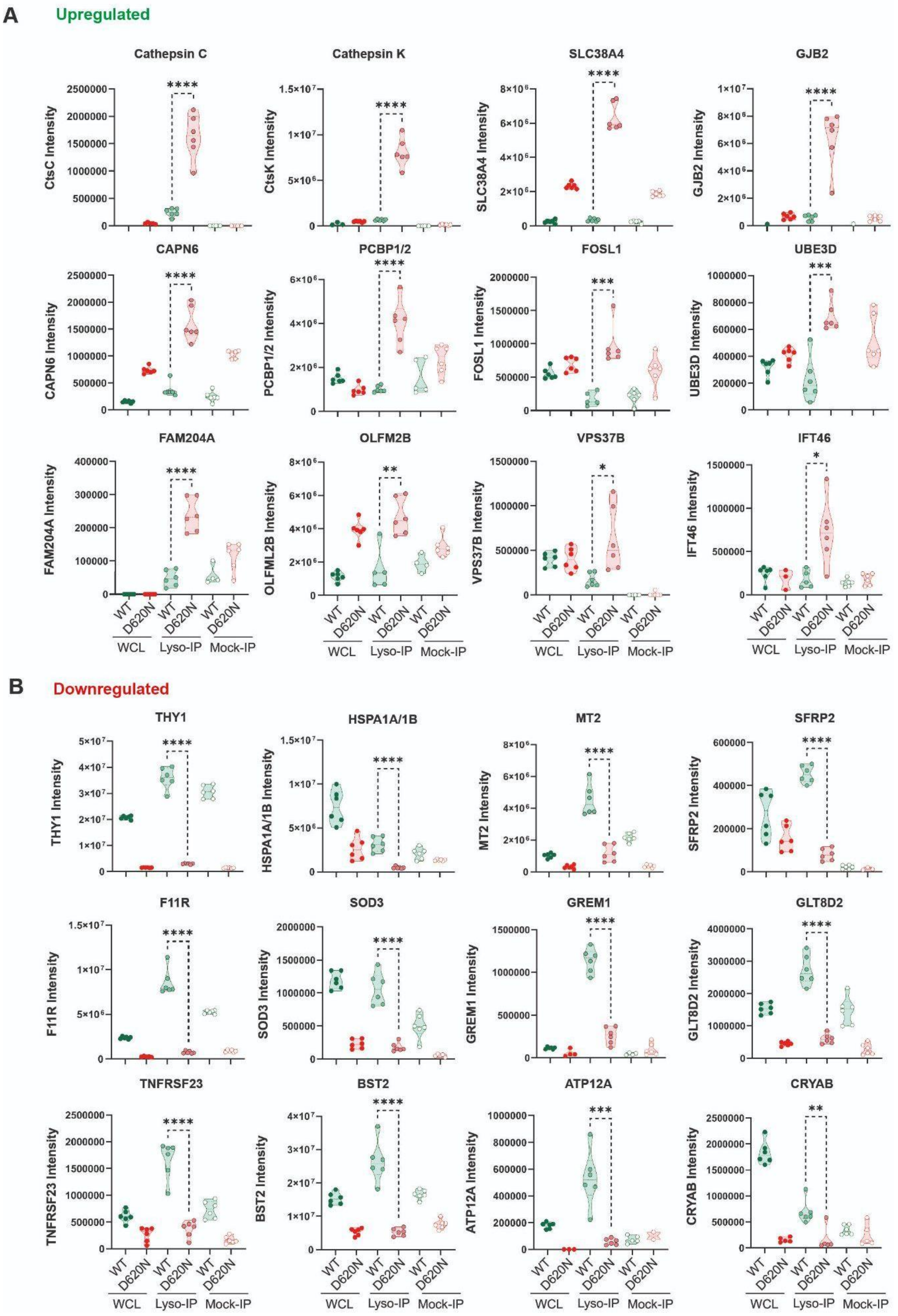

Fig. S2. Relative expression of selected proteins in wild type and D620N whole cell lysates and lysosomes. Violin plots of the indicated proteins that are most (A) upregulated or (B) downregulated in the presence of VPS35[D620N] mutation are shown. Data were analyzed using two-tailed unpaired t-test (** $p < 0.01$, *** $p < 0.001$, **** $p < 0.001$).

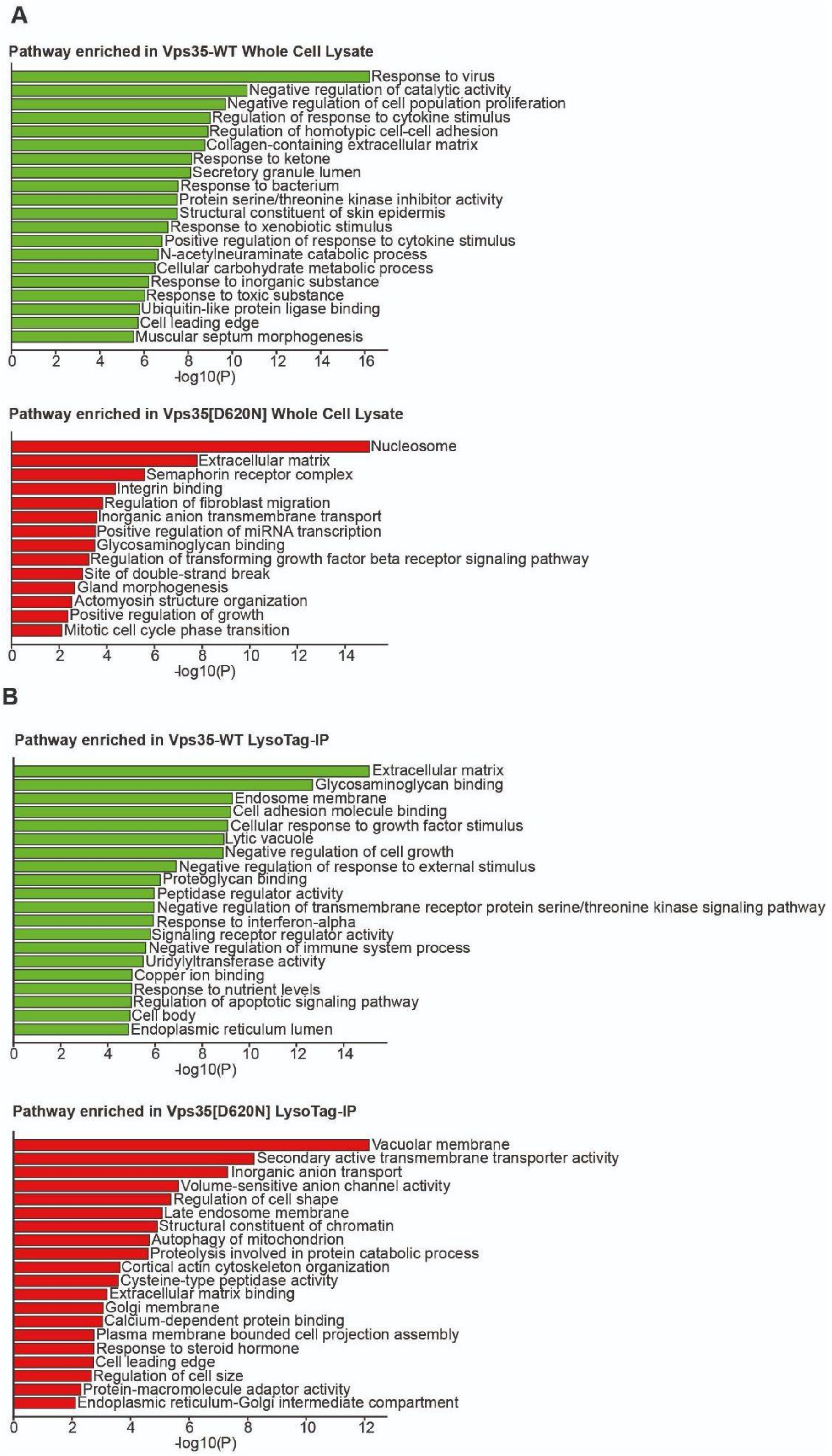

Fig. S3. Gene Ontology analysis of proteins whose levels are most affected by the D620N mutations. The GO term pathway analysis of significant down-regulated (green bar) and up-regulated proteins (red bar) observed in (A) WCL (Fig. 1C) and (B) LysoTag-IP (Fig. 1D) experiments with fold change > 1.5 and p-value <0.05 using Metascape software (RRID:SCR_016620, version 5.3) with an enrichment of p-value < 0.01.

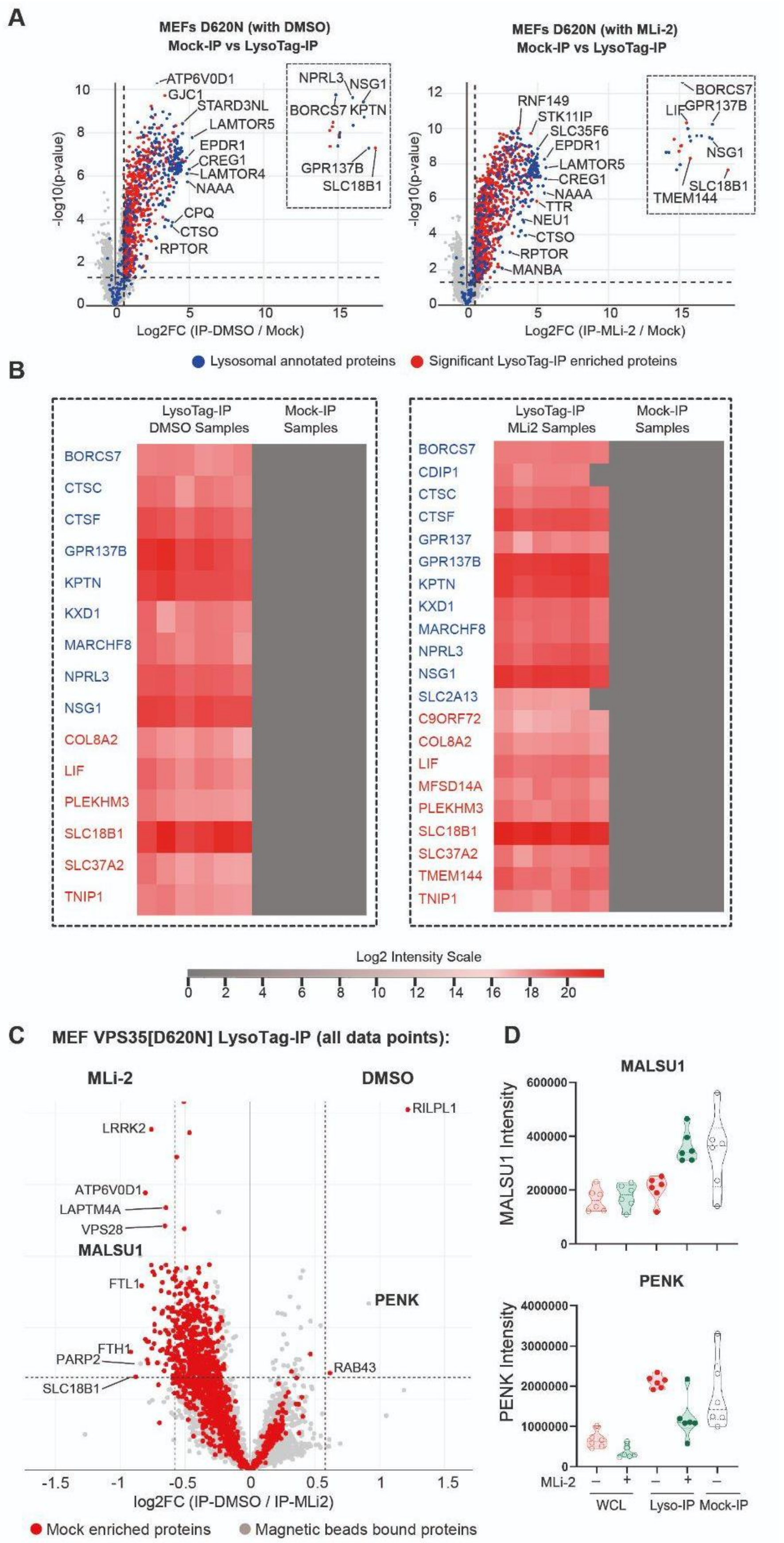

Fig. S4. Further Mass Spectrometry analysis of D60N MEFs treated ± MLi-2. (A) The volcano plots show the result of MEFs VPS35[D620N] (no LysoTag) (Mock-IP)

versus VPS35[D620N] (with DMSO) LysoTag-IP (TMEM192-3xHA) samples (left) (Curtain link: https://curtain.proteo.info/#/523b531b-eb5b-40a9-9f91-0aa7f09a002e) and MEFs VPS35[D620N] (no LysoTag) versus VPS35[D620N] (with MLi-2) LysoTag-IP samples (right) (Curtain link:https://curtain.proteo.info/#/100602aa-ca9d-4a5f-a821-4dc98b637570) (Table S4). The blue dots represent lysosomal proteins annotated in the GO terms database (GO:0005764), while the red dots represent significantly enriched proteins with fold change > 1.5 and p-value < 0.05. The black dotted line box indicates proteins confidently detected in the LysoTag-IP samples but not in the Mock-IP samples. The log2 MS intensities of these proteins are shown in Supplementary Figure (B). (C) The volcano plot (Curtain link: https://curtain.proteo.info/#/e10d5d9a-3214-4a5d-88cb-7ca1b1460e1c) shows the proteome changes of all detected proteins in MEFs VPS35[D620N] MLi-2 versus DMSO LysoTag-IP experiments. The red dots represent mock-enriched proteins with fold change > 1.5 and p-value < 0.05. The gray dots represent proteins bound to magnetic beads, which do not show significant enrichment. (D) The raw MS intensities of two non-specific proteins that associate with the magnetic beads (MALSU1 and PENK) were shown for reference.

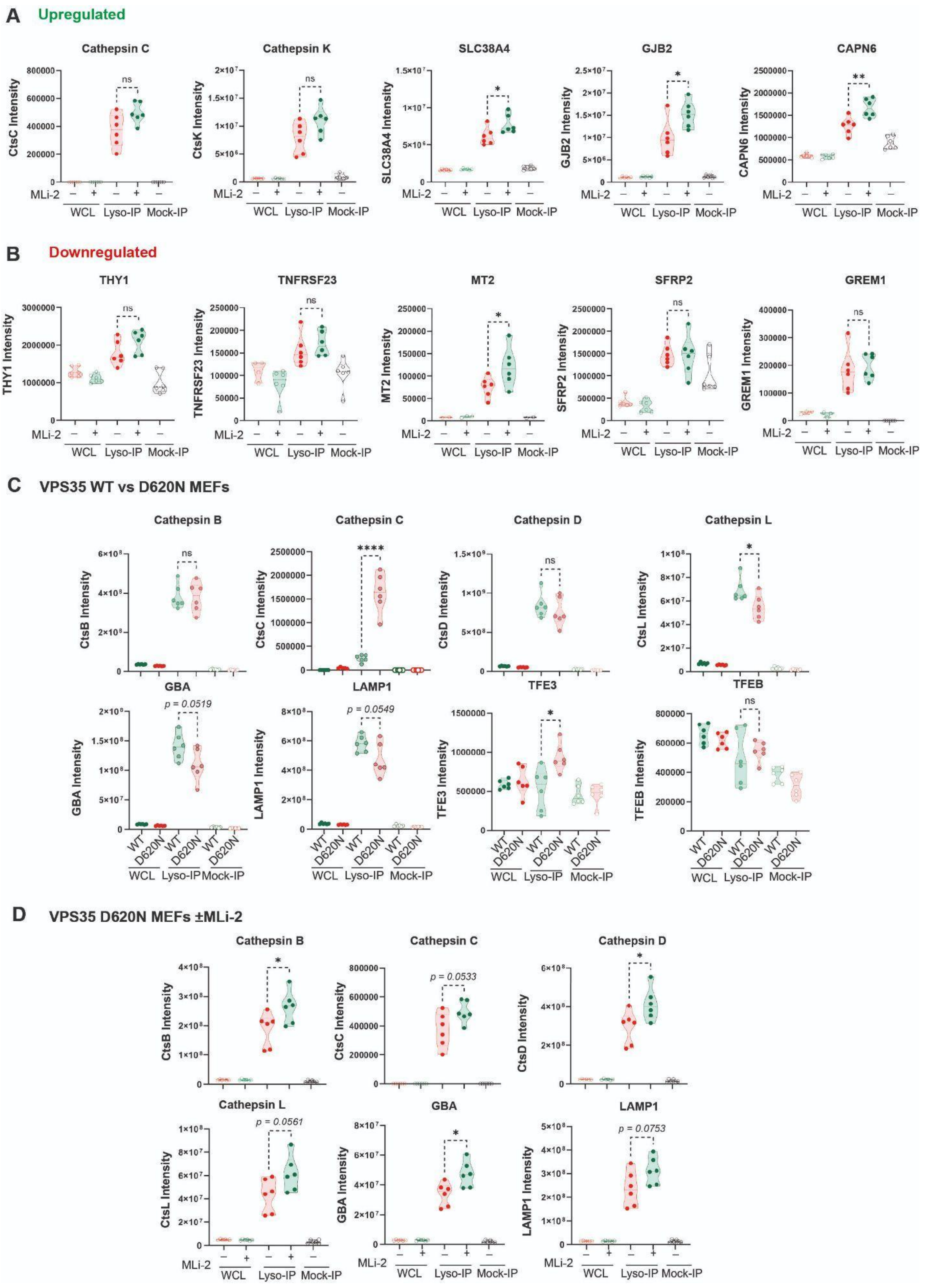

Fig. S5. Relative levels of selected proteins in lysate and lysosomes of wild type and D620N MEFs and impact of MLi-2. (A to D) Violin plots of the indicated proteins from MS data presented in Fig. 1, A and C; and Fig. 2, B and D). Data were analyzed using two-tailed unpaired t-test (** p< 0.01, *** p< 0.001, **** p< 0.001).

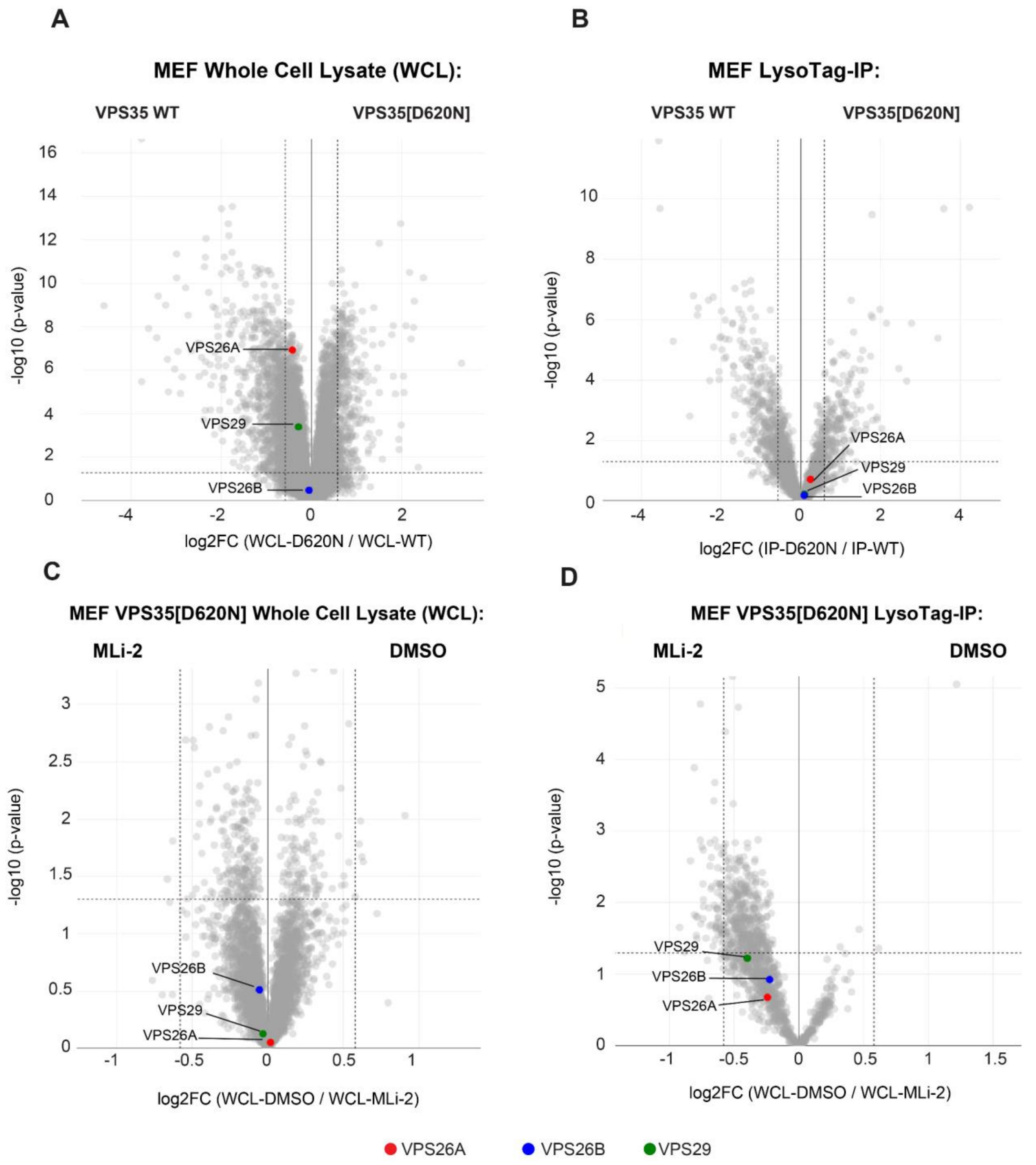

Fig. S6 Enrichment of proteins related to the retromer complex. Volcano-plots show the enrichment of VPS26A, VPS26B, and VPS29, proteins which are the part of the retromer complex apart from VPS35. Comparison between the (A) Whole cell lysate (WCL) and (B) LysoTag-IP from the VPS35 WT and D620N MEFs. Comparison between the (C) Whole cell lysate (WCL) and (D) LysoTag-IP from the VPS35 D620N MEFs treated with ±MLi-2.

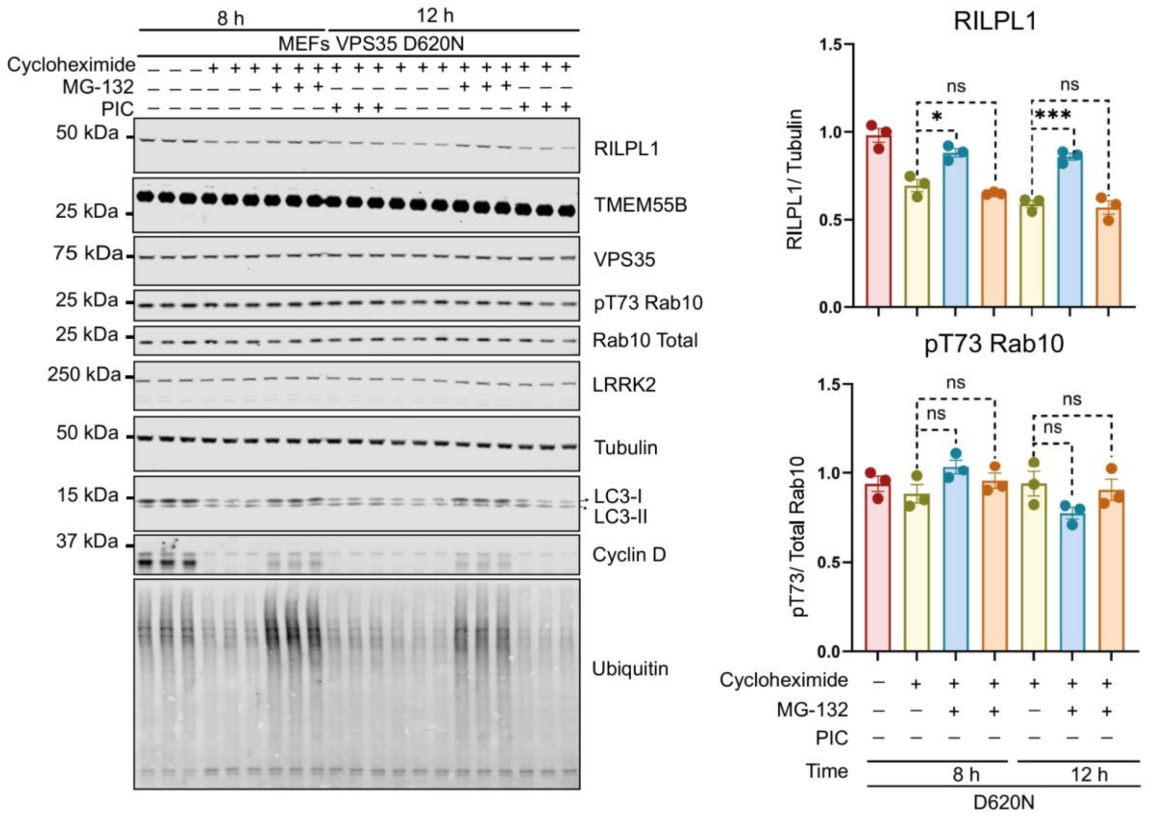

Fig. S7 Proteasome mediated degradation of RILPL1. VPS35 D620N MEFs were treated with either 50 µg/mL of cycloheximide alone, or cycloheximide+10 µM MG-132, or cycloheximide + protease inhibitor cocktails (PIC - 5 µM of E64D + 10 µM of Leupeptin + 10 µM of pepstatin A) for 8 h and 12 h prior to lysis. Lysates were subjected to quantitative immunoblot analysis using the LI-COR Odyssey CLx Western Blot imaging and the indicated antibodies.Technical replicates represent cell extracts obtained from three different dishes of cells. Quantitation of immunoblotting data (performed using ImageStudioLite software version 5.2.5, RRID:SCR_013715) is shown as mean ± SEM. Data were analyzed using two-tailed unpaired student's *t*-test (*$p < 0.05$, ***$p < 0.001$).

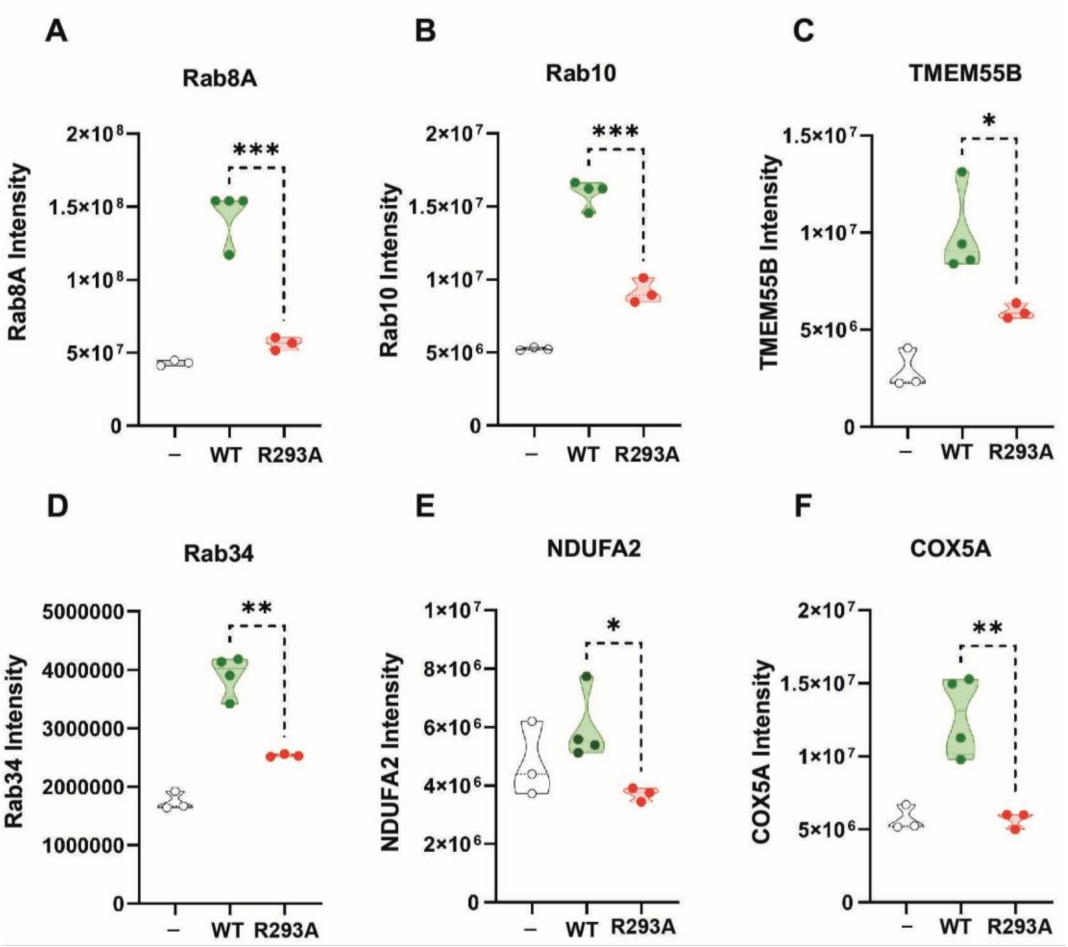

Fig. S8. Relative levels of RILPL1 interacting proteins derived from Mass spectrometry data. (A to F) Violin plots of the indicated proteins from MS data presented in Fig. 5C. Data were analyzed using two-tailed unpaired t-test (*p< 0.05, ** p< 0.01, *** p< 0.001).

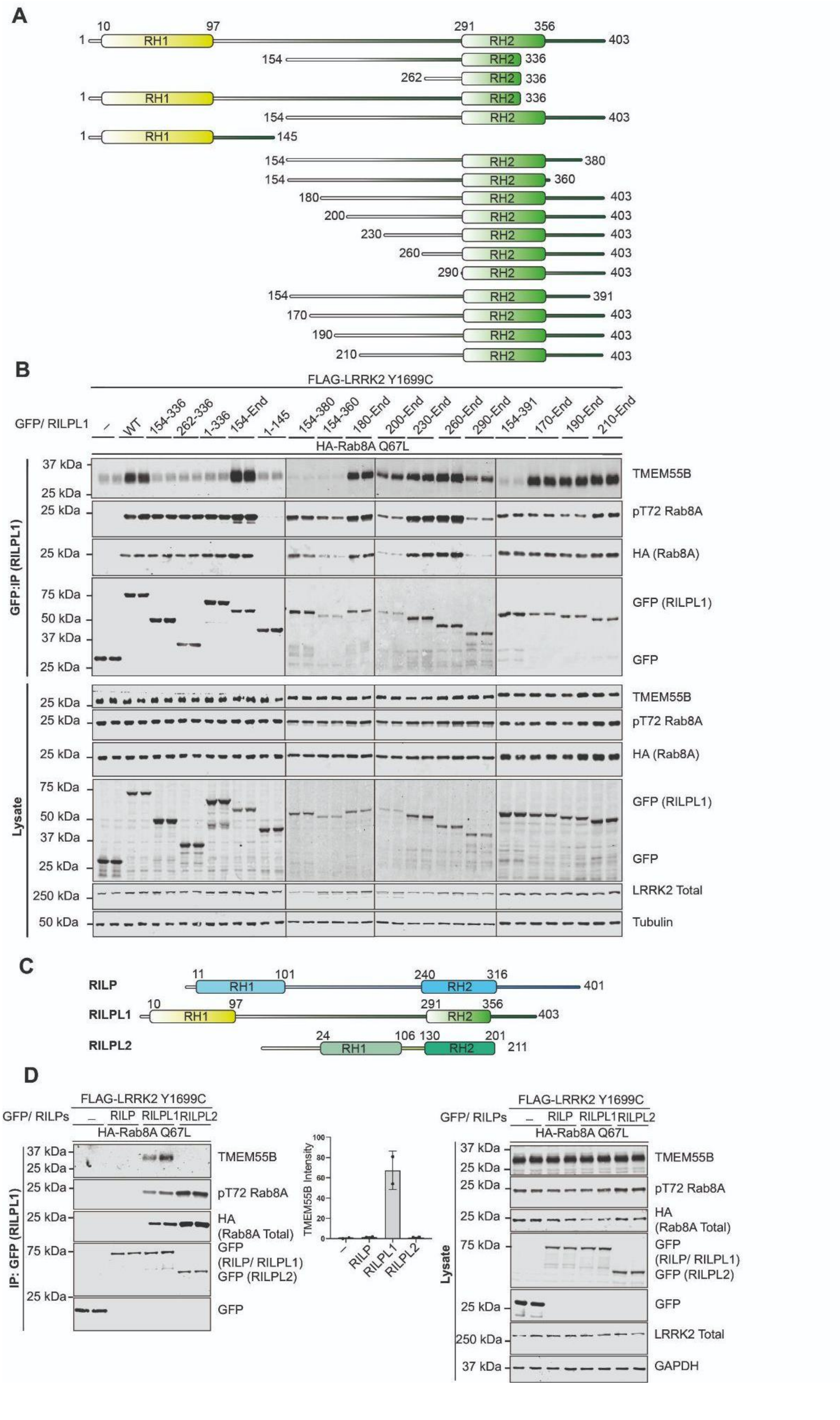

Fig. S9. Further confirmation that RILPL1 interacts with TMEM55B but not RILP or RILPL2. (A) Domain structure of full length and truncated RILPL1 mutants. (B) HEK293 cells were transiently transfected with the indicated proteins and lysed 24h

post transfection. GFP-RILPL1 immunoprecipitations (top panel) or cell extracts (lower panel) were subjected to quantitative immunoblot analysis using the LI-COR Odyssey CLx Western Blot imaging system and indicated antibodies. Quantitation of immunoblotting data (performed using ImageStudioLite software version 5.2.5, RRID:SCR_013715) is shown as mean ± SEM. (C) Domain structure of full length RILP, RILPL1 and RILP2. (D) As in (B).

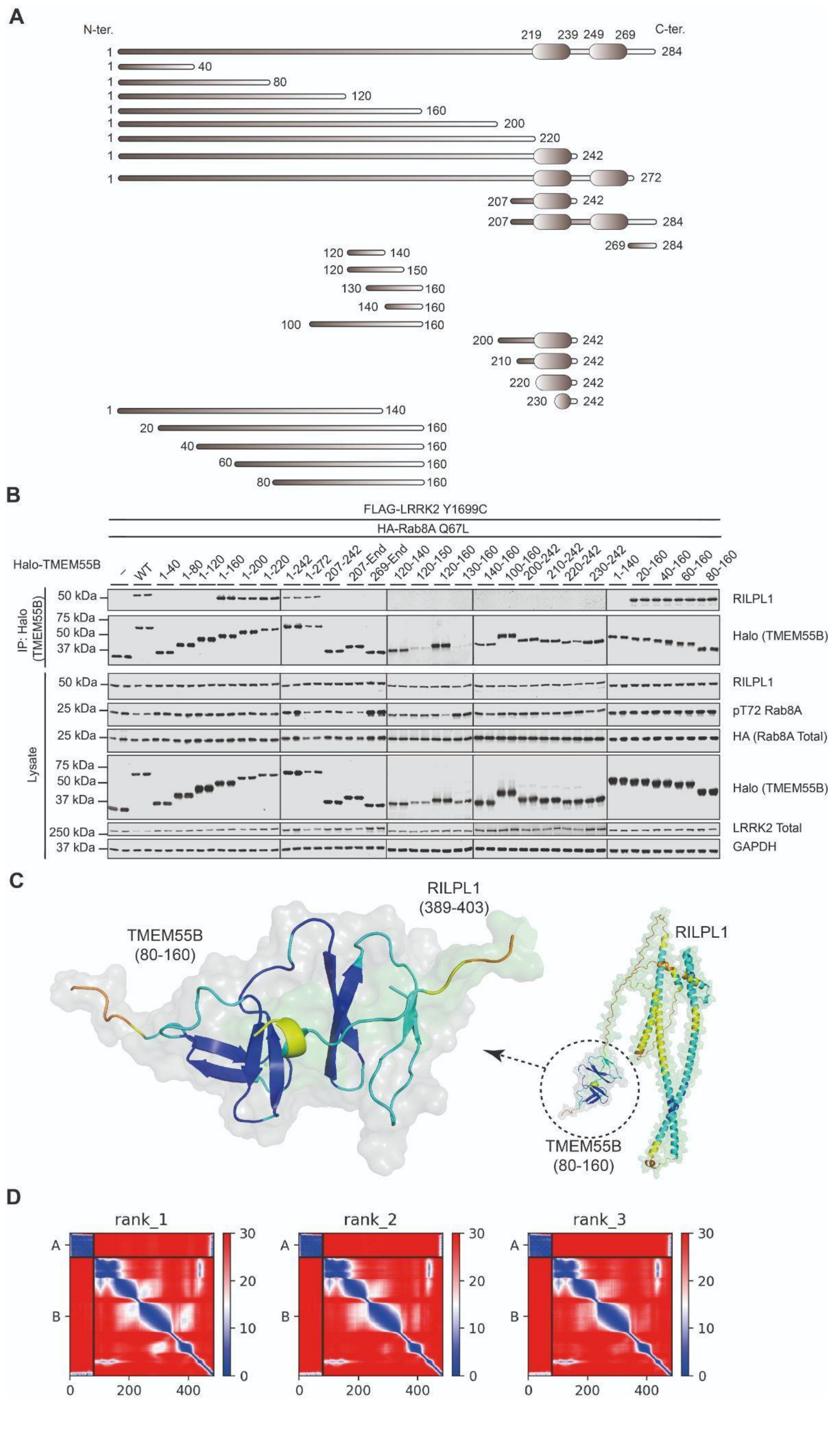

Fig. S10. Confirmation that the TMEM55B Conserved domain binds RILPL1 and the AlphaFold2 model of this interaction. (A) Domain structure of full length and truncated mutants of TMEM55B used in this study. (B) HEK293 cells were transiently transfected with the indicated proteins and lysed 24h post transfection. Halo-

TMEM55B immunoprecipitations (top panel) or cell extracts (lower panel) were subjected to quantitative immunoblot analysis using the LI-COR Odyssey CLx Western Blot imaging system and indicated antibodies. (C) Top ranked model confidence scores (pLDDT) overlay on the structure (Dark blue: pLDDT>90; Blue: 90>pLDDT>70; Yellow: 70>pLDDT>50; Orange: pLDDT<50). (D) PAE matrix plots for the top 3 models where A = RILPL1 (389-403), and B = TMEM55B (80-160).

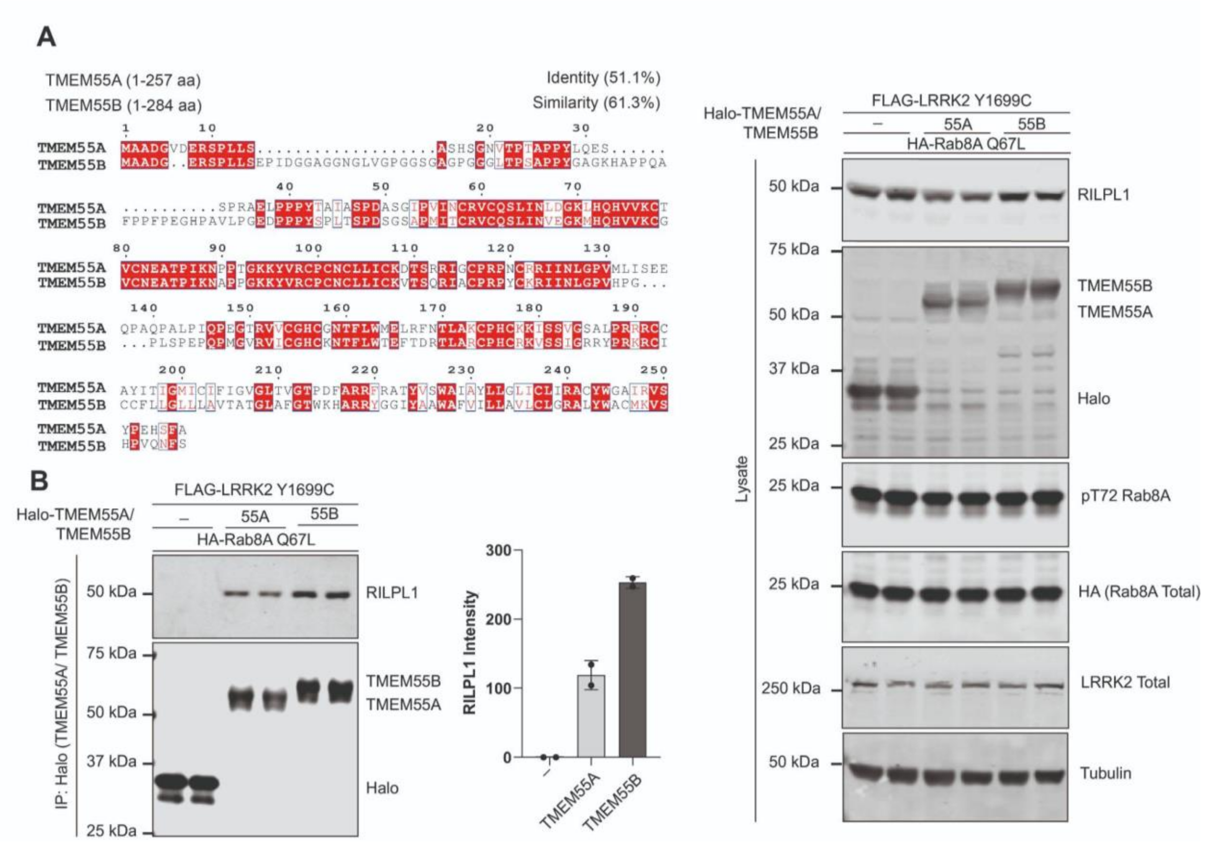

Fig. S11. Identity and similarity of TMEM55A and TMEM55B and their interaction with RILPL1. (A) Multiple sequence alignment of TMEM55A and TMEM55B using Clustal Omega (https://www.ebi.ac.uk/Tools/msa/clustalo/) and ESPript (https://espript.ibcp.fr/ESPript/cgi-bin/ESPript.cgi) (82) (B) HEK293 cells were transiently transfected with the indicated proteins and lysed 24h post transfection. Halo-TMEM55A and -TMEM55B immunoprecipitations and whole cell lysates were subjected to quantitative immunoblot analysis using the LI-COR Odyssey CLx Western Blot imaging system and indicated antibodies.

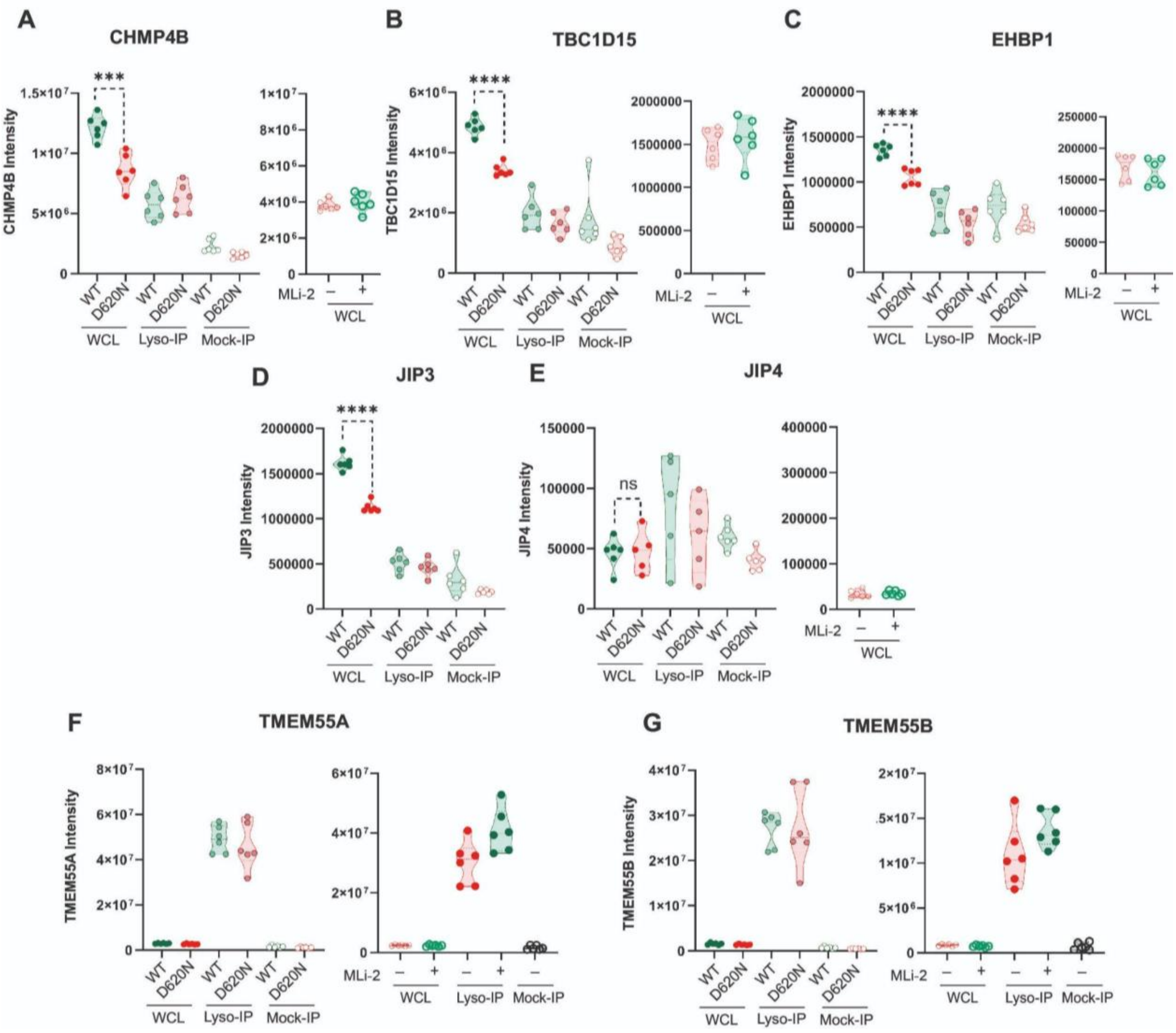

Fig. S12. Relative levels of proteins in D620N Lysates and lysosomes that have been reported to be recruited to the lysosome in LLOMe treated cells. (A to G) Violin plots of the indicated proteins from MS data presented in Figure 1 (data on left panels) and Figure 2 (data on right panels). Data were analyzed using two-tailed unpaired *t*-test (*** p< 0.001, **** p< 0.0001).

Legends for Tables S4 to S6 that are Excel sheets submitted separately as auxiliary supplementary files

Table S4. Search result of DIA MS data: VPS35 WT vs D620N WCL and LysoTag-IP

Table S5. Search result of DIA MS data: VPS35 D620N ± MLi-2 WCL and LysoTag-IP

Table S6. Search result of TMT MS data: RILPL1 WT vs R293A GFP-IP

REFERENCES AND NOTES

1. D. R. Alessi, E. Sammler, LRRK2 kinase in Parkinson's disease. *Science* **360**, 36–37 (2018).

2. E. Tolosa, M. Vila, C. Klein, O. Rascol, LRRK2 in Parkinson disease: Challenges of clinical trials. *Nat. Rev. Neurol.* **16**, 97–107 (2020).

3. M. K. Herrick, M. G. Tansey, Is LRRK2 the missing link between inflammatory bowel disease and Parkinson's disease? *NPJ Parkinsons Dis.* **7**, 26 (2021).

4. K. Y. Hui, H. Fernandez-Hernandez, J. Hu, A. Schaffner, N. Pankratz, N. Y. Hsu, L. S. Chuang, S. Carmi, N. Villaverde, X. Li, M. Rivas, A. P. Levine, X. Bao, P. R. Labrias, T. Haritunians, D. Ruane, K. Gettler, E. Chen, D. Li, E. R. Schiff, N. Pontikos, N. Barzilai, S. R. Brant, S. Bressman, A. S. Cheifetz, L. N. Clark, M. J. Daly, R. J. Desnick, R. H. Duerr, S. Katz, T. Lencz, R. H. Myers, H. Ostrer, L. Ozelius, H. Payami, Y. Peter, J. D. Rioux, A. W. Segal, W. K. Scott, M. S. Silverberg, J. M. Vance, I. Ubarretxena-Belandia, T. Foroud, G. Atzmon, I. Pe'er, Y. Ioannou, D. P. B. McGovern, Z. Yue, E. E. Schadt, J. H. Cho, I. Peter, Functional variants in the *LRRK2* gene confer shared effects on risk for Crohn's disease and Parkinson's disease. *Sci. Transl. Med.* **10**, eaai7795 (2018).

5. M. Taylor, D. R. Alessi, Advances in elucidating the function of leucine-rich repeat protein kinase-2 in normal cells and Parkinson's disease. *Curr. Opin. Cell Biol.* **63**, 102–113 (2020).

6. M. Steger, F. Diez, H. S. Dhekne, P. Lis, R. S. Nirujogi, O. Karayel, F. Tonelli, T. N. Martinez, E. Lorentzen, S. R. Pfeffer, D. R. Alessi, M. Mann, Systematic proteomic analysis of LRRK2-mediated Rab GTPase phosphorylation establishes a connection to ciliogenesis. *eLife* **6**, e31012 (2017).

7. M. Steger, F. Tonelli, G. Ito, P. Davies, M. Trost, M. Vetter, S. Wachter, E. Lorentzen, G. Duddy, S. Wilson, M. A. Baptista, B. K. Fiske, M. J. Fell, J. A. Morrow, A. D. Reith, D. R. Alessi, M. Mann, Phosphoproteomics reveals that Parkinson's disease kinase LRRK2 regulates a subset of Rab GTPases. *eLife* **5**, e12813 (2016).

8. S. R. Pfeffer, Rab GTPases: Master regulators that establish the secretory and endocytic pathways. *Mol. Biol. Cell* **28**, 712–715 (2017).

9. K. Berndsen, P. Lis, W. M. Yeshaw, P. S. Wawro, R. S. Nirujogi, M. Wightman, T. Macartney, M. Dorward, A. Knebel, F. Tonelli, S. R. Pfeffer, D. R. Alessi, PPM1H phosphatase counteracts LRRK2 signaling by selectively dephosphorylating Rab proteins. *eLife* **8**, e50416 (2019).

10. D. Waschbusch, K. Berndsen, P. Lis, A. Knebel, Y. P. Lam, D. R. Alessi, A. R. Khan, Structural basis for the specificity of PPM1H phosphatase for Rab GTPases. *EMBO Rep.* **22**, e52675 (2021).

11. D. Waschbüsch, E. Purlyte, P. Pal, E. McGrath, D. R. Alessi, A. R. Khan, Structural basis for Rab8a recruitment of RILPL2 via LRRK2 phosphorylation of switch 2. *Structure* **28**, 406–417.e6 (2020).

12. L. Bonet-Ponce, A. Beilina, C. D. Williamson, E. Lindberg, J. H. Kluss, S. Saez-Atienzar, N. Landeck, R. Kumaran, A. Mamais, C. K. E. Bleck, Y. Li, M. R. Cookson, LRRK2 mediates tubulation and vesicle sorting from lysosomes. *Sci. Adv.* **6**, eabb2454 (2020).

13. H. S. Dhekne, I. Yanatori, R. C. Gomez, F. Tonelli, F. Diez, B. Schule, M. Steger, D. R. Alessi, S. R. Pfeffer, A pathway for Parkinson's disease LRRK2 kinase to block primary cilia and Sonic hedgehog signaling in the brain. *Elife* **7**, e40202 (2018).

14. Y. Sobu, P. S. Wawro, H. S. Dhekne, W. M. Yeshaw, S. R. Pfeffer, Pathogenic LRRK2 regulates ciliation probability upstream of tau tubulin kinase 2 via Rab10 and RILPL1 proteins. *Proc. Natl. Acad. Sci. U.S.A.* **118**, e2005894118 (2021).

15. E. Purlyte, H. S. Dhekne, A. R. Sarhan, R. Gomez, P. Lis, M. Wightman, T. N. Martinez, F. Tonelli, S. R. Pfeffer, D. R. Alessi, Rab29 activation of the Parkinson's disease-associated LRRK2 kinase. *EMBO J.* **37**, 1–18 (2018).

16. Z. Liu, N. Bryant, R. Kumaran, A. Beilina, A. Abeliovich, M. R. Cookson, A. B. West, LRRK2 phosphorylates membrane-bound Rabs and is activated by GTP-bound Rab7L1 to promote recruitment to the trans-Golgi network. *Hum. Mol. Genet.* **27**, 385–395 (2018).

17. E. McGrath, D. Waschbüsch, B. M. Baker, A. R. Khan, LRRK2 binds to the Rab32 subfamily in a GTP-dependent manner via its armadillo domain. *Small GTPases* **12**, 133–146 (2021).

18. E. G. Vides, A. Adhikari, C. Y. Chiang, P. Lis, E. Purlyte, C. Limouse, J. L. Shumate, E. Spinola-Lasso, H. S. Dhekne, D. R. Alessi, S. R. Pfeffer, A feed-forward pathway drives LRRK2 kinase membrane recruitment and activation. *eLife* **11**, e79771 (2022).

19. H. Zhu, F. Tonelli, D. R. Alessi, J. Sun, Structural basis of human LRRK2 membrane recruitment and activation. bioRxiv 2022.04.26.489605 [**Preprint**]. 26 April 2022. https://doi.org/10.1101/2022.04.26.489605.

20. H. S. Dhekne, F. Tonelli, W. M. Yeshaw, C. Y. Chiang, C. Limouse, E. Jaimon, E. Purlyte, D. R. Alessi, S. R. Pfeffer, Genome-wide screen reveals Rab12 GTPase as a critical activator of pathogenic LRRK2 kinase. bioRxiv 2023.02.17.529028 [**Preprint**]. 18 February 2023. https://doi.org/10.1101/2023.02.17.529028.

21. V. V. Bondar, X. Wang, O. B. Davis, M. T. Maloney, M. Agam, M. Y. Chin, A. C.-N. Ho, D. Joy, J. W. Lewcock, G. D. Paolo, R. G. Thorne, Z. K. Sweeney, A. G. Henry, Rab12 regulates LRRK2 activity by promoting its localization to lysosomes. bioRxiv 2023.02.21.529466 [**Preprint**]. 22 February 2023. https://doi.org/10.1101/2023.02.21.529466.

22. G. K. Tofaris, Lysosome-dependent pathways as a unifying theme in Parkinson's disease. *Mov. Disord.* **27**, 1364–1369 (2012).

23. A. Navarro-Romero, M. Montpeyo, M. Martinez-Vicente, The emerging role of the lysosome in Parkinson's disease. *Cell* **9**, 2399 (2020).

24. E. Sidransky, M. A. Nalls, J. O. Aasly, J. Aharon-Peretz, G. Annesi, E. R. Barbosa, A. Bar-Shira, D. Berg, J. Bras, A. Brice, C. M. Chen, L. N. Clark, C. Condroyer, E. V. De Marco, A. Durr, M. J. Eblan, S. Fahn, M. J. Farrer, H. C. Fung, Z. Gan-Or, T. Gasser, R. Gershoni-Baruch, N. Giladi, A. Griffith, T. Gurevich, C. Januario, P. Kropp, A. E. Lang, G. J. Lee-Chen, S. Lesage, K. Marder, I. F. Mata, A. Mirelman, J. Mitsui, I. Mizuta, G. Nicoletti, C. Oliveira, R. Ottman, A. Orr-Urtreger, L. V. Pereira, A. Quattrone, E. Rogaeva, A. Rolfs, H. Rosenbaum, R. Rozenberg, A. Samii, T. Samaddar, C. Schulte, M. Sharma, A. Singleton, M. Spitz, E. K. Tan, N. Tayebi, T. Toda, A. R. Troiano, S. Tsuji, M. Wittstock, T. G. Wolfsberg, Y. R. Wu, C. P. Zabetian, Y. Zhao, S. G. Ziegler, Multicenter analysis of glucocerebrosidase mutations in Parkinson's disease. *N. Engl. J. Med.* **361**, 1651–1661 (2009).

25. S. van Veen, S. Martin, C. Van den Haute, V. Benoy, J. Lyons, R. Vanhoutte, J. P. Kahler, J. P. Decuypere, G. Gelders, E. Lambie, J. Zielich, J. V. Swinnen, W. Annaert, P. Agostinis, B. Ghesquiere, S. Verhelst, V. Baekelandt, J. Eggermont, P. Vangheluwe, ATP13A2 deficiency disrupts lysosomal polyamine export. *Nature* **578**, 419–424 (2020).

26. M. Hu, P. Li, C. Wang, X. Feng, Q. Geng, W. Chen, M. Marthi, W. Zhang, C. Gao, W. Reid, J. Swanson, W. Du, R. I. Hume, H. Xu, Parkinson's disease-risk protein TMEM175 is a proton-activated proton channel in lysosomes. *Cell* **185**, 2292–2308.e20 (2022).

27. M. Madureira, N. Connor-Robson, R. Wade-Martins, LRRK2: Autophagy and lysosomal activity. *Front. Neurosci.* **14**, 498 (2020).

28. C. A. Boecker, J. Goldsmith, D. Dou, G. G. Cajka, E. L. F. Holzbaur, Increased LRRK2 kinase activity alters neuronal autophagy by disrupting the axonal transport of autophagosomes. *Curr. Biol.* **31**, 2140–2154.e6 (2021).

29. C. A. Boecker, E. L. F. Holzbaur, Hyperactive LRRK2 kinase impairs the trafficking of axonal autophagosomes. *Autophagy* **17**, 2043–2045 (2021).

30. F. Singh, A. R. Prescott, P. Rosewell, G. Ball, A. D. Reith, I. G. Ganley, Pharmacological rescue of impaired mitophagy in Parkinson's disease-related LRRK2 G2019S knock-in mice. *eLife* **10**, e67604 (2021).

31. S. Herbst, P. Campbell, J. Harvey, E. M. Bernard, V. Papayannopoulos, N. W. Wood, H. R. Morris, M. G. Gutierrez, LRRK2 activation controls the repair of damaged endomembranes in macrophages. *EMBO J.* **39**, e104494 (2020).

32. A. F. Kalogeropulou, J. B. Freemantle, P. Lis, E. G. Vides, N. K. Polinski, D. R. Alessi, Endogenous Rab29 does not impact basal or stimulated LRRK2 pathway activity. *Biochem. J.* **477**, 4397–4423 (2020).

33. N. Yadavalli, S. M. Ferguson, LRRK2 suppresses lysosome degradative activity in macrophages and microglia through MiT-TFE transcription factor inhibition. *Proc. Natl. Acad. Sci. U.S.A.* **120**, e2303789120 (2023).

34. R. N. Alcalay, F. Hsieh, E. Tengstrand, S. Padmanabhan, M. Baptista, C. Kehoe, S. Narayan, A. K. Boehme, K. Merchant, Higher urine bis(monoacylglycerol)phosphate levels in LRRK2 G2019S mutation carriers: Implications for therapeutic development. *Mov. Disord.* **35**, 134–141 (2020).

35. M. T. Maloney, X. Wang, R. Ghosh, S. V. Andrews, R. Maciuca, S. T. Masoud, R. M. Caprioli, J. Chen, C.-L. Chiu, S. S. Davis, A. C.-N. Ho, H. N. Nguyen, N. E. Propson, M. L. Reyzer, O. B. Davis, M. C. Deen, S. Zhu, G. Di Paolo, D. J. Vocadlo, A. A. Estrada, J. de Vicente, J. W. Lewcock, A. Arguello, J. H. Suh, S. Huntwork-Rodriguez, A. G. Henry, LRRK2 kinase activity regulates Parkinson's disease-relevant lipids at the lysosome. bioRxiv 2022.12.19.521070 [**Preprint**]. 19 December 2022. https://doi.org/10.1101/2022.12.19.521070.

36. A. Zimprich, A. Benet-Pages, W. Struhal, E. Graf, S. H. Eck, M. N. Offman, D. Haubenberger, S. Spielberger, E. C. Schulte, P. Lichtner, S. C. Rossle, N. Klopp, E. Wolf, K. Seppi, W. Pirker, S. Presslauer, B. Mollenhauer, R. Katzenschlager, T. Foki, C. Hotzy, E. Reinthaler, A. Harutyunyan, R. Kralovics, A. Peters, F. Zimprich, T. Brucke, W. Poewe, E. Auff, C. Trenkwalder, B. Rost, G. Ransmayr, J. Winkelmann, T. Meitinger, T. M. Strom, A mutation in VPS35, encoding a subunit of the retromer complex, causes late-onset Parkinson disease. *Am. J. Hum. Genet.* **89**, 168–175 (2011).

37. C. Vilarino-Guell, C. Wider, O. A. Ross, J. C. Dachsel, J. M. Kachergus, S. J. Lincoln, A. I. Soto-Ortolaza, S. A. Cobb, G. J. Wilhoite, J. A. Bacon, B. Behrouz, H. L. Melrose, E. Hentati, A. Puschmann, D. M. Evans, E. Conibear, W. W. Wasserman, J. O. Aasly, P. R. Burkhard, R. Djaldetti, J. Ghika, F. Hentati, A. Krygowska-Wajs, T. Lynch, E. Melamed, A. Rajput, A. H. Rajput, A. Solida, R. M. Wu, R. J. Uitti, Z. K. Wszolek, F. Vingerhoets, M. J. Farrer, VPS35 mutations in Parkinson disease. *Am. J. Hum. Genet.* **89**, 162–167 (2011).

38. S. A. Small, K. Kent, A. Pierce, C. Leung, M. S. Kang, H. Okada, L. Honig, J. P. Vonsattel, T. W. Kim, Model-guided microarray implicates the retromer complex in Alzheimer's disease. *Ann. Neurol.* **58**, 909–919 (2005).

39. R. Mir, F. Tonelli, P. Lis, T. Macartney, N. K. Polinski, T. N. Martinez, M.-Y. Chou, A. J. M. Howden, T. König, C. Hotzy, I. Milenkovic, T. Brücke, A. Zimprich, E. Sammler, D. R. Alessi, The Parkinson's disease VPS35[D620N] mutation enhances LRRK2-mediated Rab protein phosphorylation in mouse and human. *Biochem. J.* **475**, 1861–1883 (2018).

40. R. S. Nirujogi, F. Tonelli, M. Taylor, P. Lis, A. Zimprich, E. Sammler, D. R. Alessi, Development of a multiplexed targeted mass spectrometry assay for LRRK2-phosphorylated Rabs and Ser^{910}/Ser^{935} biomarker sites. *Biochem. J.* **478**, 299–326 (2021).

41. T. S. Gomez, D. D. Billadeau, A FAM21-containing WASH complex regulates retromer-dependent sorting. *Dev. Cell* **17**, 699–711 (2009).

42. Y. Cui, Z. Yang, R. D. Teasdale, The functional roles of retromer in Parkinson's disease. *FEBS Lett.* **592**, 1096–1112 (2017).

43. M. Abu-Remaileh, G. A. Wyant, C. Kim, N. N. Laqtom, M. Abbasi, S. H. Chan, E. Freinkman, D. M. Sabatini, Lysosomal metabolomics reveals V-ATPase- and mTOR-dependent regulation of amino acid efflux from lysosomes. *Science* **358**, 807–813 (2017).

44. V. Demichev, C. B. Messner, S. I. Vernardis, K. S. Lilley, M. Ralser, DIA-NN: Neural networks and interference correction enable deep proteome coverage in high throughput. *Nat. Methods* **17**, 41–44 (2020).

45. T. K. Phung, K. Berndsen, T. L. C. H. B. Phan, M. M. K. Muqit, D. R. Alessi, R. S. Nirujogi, CURTAIN—A unique web-based tool for exploration and sharing of MS-based proteomics data. bioRxiv 2023.07.25.550405 [**Preprint**]. 25 July 2023. https://doi.org/10.1101/2023.07.25.550405.

46. Y. Zhou, B. Zhou, L. Pache, M. Chang, A. H. Khodabakhshi, O. Tanaseichuk, C. Benner, S. K. Chanda, Metascape provides a biologist-oriented resource for the analysis of systems-level datasets. *Nat. Commun.* **10**, 1523 (2019).

47. R. Fasimoye, W. Dong, R. S. Nirujogi, E. S. Rawat, M. Iguchi, K. Nyame, T. K. Phung, E. Bagnoli, A. R. Prescott, D. R. Alessi, M. Abu-Remaileh, Golgi-IP, a tool for multimodal analysis of Golgi molecular content. *Proc. Natl. Acad. Sci. U.S.A.* **120**, e2219953120 (2023).

48. M. J. Fell, C. Mirescu, K. Basu, B. Cheewatrakoolpong, D. E. DeMong, J. M. Ellis, L. A. Hyde, Y. Lin, C. G. Markgraf, H. Mei, M. Miller, F. M. Poulet, J. D. Scott, M. D. Smith, Z. Yin, X. Zhou, E. M. Parker, M. E. Kennedy, J. A. Morrow, MLi-2, a potent, selective, and centrally active compound for exploring the therapeutic potential and safety of LRRK2 kinase inhibition. *J. Pharmacol. Exp. Ther.* **355**, 397–409 (2015).

49. K. Ito, M. Araki, Y. Katai, Y. Nishimura, S. Imotani, H. Inoue, G. Ito, T. Tomita, Pathogenic LRRK2 compromises the subcellular distribution of lysosomes in a Rab12-RILPL1-dependent manner. *FASEB J.* **37**, e22930 (2023).

50. A. J. Lara Ordonez, R. Fasiczka, Y. Naaldijk, S. Hilfiker, Rab GTPases in Parkinson's disease: A primer. *Essays Biochem.* **65**, 961–974 (2021).

51. G. Ito, K. Katsemonova, F. Tonelli, P. Lis, M. A. Baptista, N. Shpiro, G. Duddy, S. Wilson, P. W. Ho, S. L. Ho, A. D. Reith, D. R. Alessi, Phos-tag analysis of Rab10 phosphorylation by LRRK2: A powerful assay for assessing kinase function and inhibitors. *Biochem. J.* **473**, 2671–2685 (2016).

52. R. Willett, J. A. Martina, J. P. Zewe, R. Wills, G. R. V. Hammond, R. Puertollano, TFEB regulates lysosomal positioning by modulating TMEM55B expression and JIP4 recruitment to lysosomes. *Nat. Commun.* **8**, 1580 (2017).

53. J. Jumper, R. Evans, A. Pritzel, T. Green, M. Figurnov, O. Ronneberger, K. Tunyasuvunakool, R. Bates, A. Zidek, A. Potapenko, A. Bridgland, C. Meyer, S. A. A. Kohl, A. J. Ballard, A. Cowie, B. Romera-Paredes, S. Nikolov, R. Jain, J. Adler, T. Back, S. Petersen, D. Reiman, E. Clancy, M. Zielinski, M. Steinegger, M. Pacholska, T. Berghammer, S. Bodenstein, D. Silver, O. Vinyals, A. W. Senior, K. Kavukcuoglu, P. Kohli, D. Hassabis, Highly accurate protein structure prediction with AlphaFold. *Nature* **596**, 583–589 (2021).

54. I. J. McGough, F. Steinberg, D. Jia, P. A. Barbuti, K. J. McMillan, K. J. Heesom, A. L. Whone, M. A. Caldwell, D. D. Billadeau, M. K. Rosen, P. J. Cullen, Retromer binding to FAM21 and the WASH complex is perturbed by the Parkinson disease-linked VPS35(D620N) mutation. *Curr. Biol.* **24**, 1670–1676 (2014).

55. E. Zavodszky, M. N. Seaman, K. Moreau, M. Jimenez-Sanchez, S. Y. Breusegem, M. E. Harbour, D. C. Rubinsztein, Mutation in VPS35 associated with Parkinson's disease impairs WASH complex association and inhibits autophagy. *Nat. Commun.* **5**, 3828 (2014).

56. J. Follett, S. J. Norwood, N. A. Hamilton, M. Mohan, O. Kovtun, S. Tay, Y. Zhe, S. A. Wood, G. D. Mellick, P. A. Silburn, B. M. Collins, A. Bugarcic, R. D. Teasdale, The Vps35 D620N mutation linked to Parkinson's disease disrupts the cargo sorting function of retromer. *Traffic* **15**, 230–244 (2014).

57. O. Kovtun, N. Leneva, Y. S. Bykov, N. Ariotti, R. D. Teasdale, M. Schaffer, B. D. Engel, D. J. Owen, J. A. G. Briggs, B. M. Collins, Structure of the membrane-assembled retromer coat determined by cryo-electron tomography. *Nature* **561**, 561–564 (2018).

58. T. Eguchi, T. Kuwahara, M. Sakurai, T. Komori, T. Fujimoto, G. Ito, S. I. Yoshimura, A. Harada, M. Fukuda, M. Koike, T. Iwatsubo, LRRK2 and its substrate Rab GTPases are sequentially targeted onto stressed lysosomes and maintain their homeostasis. *Proc. Natl. Acad. Sci. U.S.A.* **115**, E9115–E9124 (2018).

59. T. Kuwahara, K. Funakawa, T. Komori, M. Sakurai, G. Yoshii, T. Eguchi, M. Fukuda, T. Iwatsubo, Roles of lysosomotropic agents on LRRK2 activation and Rab10 phosphorylation. bioRxiv 2020.08.25.267385 [**Preprint**]. 25 August 2020. https://doi.org/10.1101/2020.08.25.267385.

60. A. Bhattacharya, R. Mukherjee, S. K. Kuncha, M. E. Brunstein, R. Rathore, S. Junek, C. Munch, I. Dikic, A lysosome membrane regeneration pathway depends on TBC1D15 and autophagic lysosomal reformation proteins. *Nat. Cell Biol.* **25**, 685–698 (2023).

61. J. L. Daly, C. M. Danson, P. A. Lewis, S. Riccardo, L. Di Filippo, D. Cacchiarelli, S. J. Cross, K. J. Heesom, A. Ballabio, J. R. Edgar, P. J. Cullen, Multiomic approach characterises the neuroprotective role of retromer in regulating lysosomal health. bioRxiv 10.1101/2022.09.13.507260 [**Preprint**]. 15 September 2022. https://doi.org/10.1101/2022.09.13.507260.

62. A. Ungewickell, C. Hugge, M. Kisseleva, S. C. Chang, J. Zou, Y. Feng, E. E. Galyov, M. Wilson, P. W. Majerus, The identification and characterization of two phosphatidylinositol-4,5-bisphosphate 4-phosphatases. *Proc. Natl. Acad. Sci. U.S.A.* **102**, 18854–18859 (2005).

63. S. Takemasu, K. Nigorikawa, M. Yamada, G. Tsurumi, S. Kofuji, S. Takasuga, K. Hazeki, Phosphorylation of TMEM55B by Erk/MAPK regulates lysosomal positioning. *J. Biochem.* **166**, 175–185 (2019).

64. Y. Hashimoto, M. Shirane, K. I. Nakayama, TMEM55B contributes to lysosomal homeostasis and amino acid-induced mTORC1 activation. *Genes Cells* **23**, 418–434 (2018).

65. F. Tonelli, Isolation of mouse embryonic fibroblasts (MEFs) from mouse embryos at E12.5 (2023). 10.17504/protocols.io.eq2ly713qlx9/v1.

66. F. Tonelli, M. Taylor, D. R. Alessi, Organelle tag introduction in mouse embryonic fibroblasts (MEFs) (2023). dx.doi.org/10.17504/protocols.io.6qpvr456bgmk/v1.

67. R. Fasimoye, D. R. Alessi, Generation of stable cell lines via retroviral or lentiviral transduction (2022). dx.doi.org/10.17504/protocols.io.kqdg3prxpl25/v1.

68. F.Tonelli, A. F. Kalogeropulou, D. R. Alessi, Assessment of in vitro kinase activity of over-expressed and endogenous LRRK2 immunoprecipitated from cells (2021). dx.doi.org/10.17504/protocols.io.bw4bpgsn.

69. F. Tonelli, D. R. Alessi, Quantitative immunoblotting analysis of LRRK2 signalling pathway V.2 (2023). dx.doi.org/10.17504/protocols.io.ewov14znkvr2/v2.

70. C. A. Hecht, H. Dhekne, W. Yeshaw, S. R. Pfeffer, Immunofluorescence microscopy of R1441C or VPS35 D620N MEF cells (2023). dx.doi.org/10.17504/protocols.io.j8nlkw1n5l5r/v1.

71. C. A. Hecht, S. S. Khan, S. Nair, C. Y. Chiang, S. R. Pfeffer, Expansion microscopy with R1441C LRRK2 MEF cells: Visualization of Myc-RILPL1 and TMEM55B (2023). dx.doi.org/10.17504/protocols.io.ewov1o8m7lr2/v1.

72. H. Dhekne, S. R. Pfeffer, An ImageJ macro for batch processing of microscopic images prior to CellProfiler automated analysis (2023). dx.doi.org/10.17504/protocols.io.3byl4bpo8vo5/v1.

73. C. A. Hecht, S. R. Pfeffer, CellProfiler pipeline to obtain Pearson's correlation coefficients for TMEM55B or pRab10 and RILPL1 (2023). dx.doi.org/10.17504/protocols.io.rm7vzbqp5vx1/v1.

74. E. Jaimon, S. R. Pfeffer, A computational pipeline to quantify perinuclear lysosomes in fibroblasts using CellProfiler (2023). dx.doi.org/10.17504/protocols.io.81wgbxrw3lpk/v1.

75. R. Fasimoye, E. Dickie, M. Taylor, F.Tonelli, D. R. Alessi, Organelle isolation from mouse tissues expressing organelle tags (2023). dx.doi.org/10.17504/protocols.io.x54v9d61zg3e/v1

76. P. Y. Lam, M.Taylor, F.Tonelli, D. R. Alessi, Organelle isolation from mouse embryonic fibroblasts (MEFs) stably expressing organelle tags for subsequent immunoblotting or proteomic analysis (2023). dx.doi.org/10.17504/protocols.io.ewov1o627lr2/v1.

77. P. Pal, R. S. Nirujogi, F.Tonelli, D. R. Alessi, GFP immunoprecipitation and sample preparation for tandem mass tag (TMT) mass spectrometry analysis (2023). dx.doi.org/10.17504/protocols.io.eq2ly7kxqlx9/v1.

78. B. Ruprecht, J. Zecha, D. P. Zolg, B. Kuster, High pH reversed-phase micro-columns for simple, sensitive, and efficient fractionation of proteome and (TMT labeled) phosphoproteome digests. *Methods Mol. Biol.* **1550**, 83–98 (2017).

79. J. Cox, M. Mann, MaxQuant enables high peptide identification rates, individualized p.p.b.-range mass accuracies and proteome-wide protein quantification. *Nat. Biotechnol.* **26**, 1367–1372 (2008).

80. S. Tyanova, T. Temu, P. Sinitcyn, A. Carlson, M. Y. Hein, T. Geiger, M. Mann, J. Cox, The Perseus computational platform for comprehensive analysis of (prote)omics data. *Nat. Methods* **13**, 731–740 (2016).

81. H. Ashkenazy, S. Abadi, E. Martz, O. Chay, I. Mayrose, T. Pupko, N. Ben-Tal, ConSurf 2016: An improved methodology to estimate and visualize evolutionary conservation in macromolecules. *Nucleic Acids Res.* **44**, W344–W350 (2016).

82. X. Robert, P. Gouet, Deciphering key features in protein structures with the new ENDscript server. *Nucleic Acids Res.* **42**, W320–W324 (2014).
